# Reassessing Polysaccharide Responsiveness: Unveiling Limitations of Current Guidelines and Introducing the Polysaccharide Responsiveness Percentile Approach

**DOI:** 10.1007/s10875-025-01915-w

**Published:** 2025-07-25

**Authors:** Stine Fischer Fogsgaard, Sonia Todaro, Carsten Schade Larsen, Charlotte Sværke Jørgensen, Jens Magnus Bernth Jensen

**Affiliations:** 1 Department of Clinical Immunology, Aarhus University Hospital, Aarhus, Denmark; 2 Department of Infectious Diseases, Aarhus University Hospital, Aarhus, Denmark; 3 Statens Serum Institut, Virus and Microbiological Preparedness, Copenhagen, Denmark; 4 Department of Molecular Medicine, Aarhus University Hospital, Aarhus, Denmark

**Keywords:** Antibody deficiency, Clinical guidelines, Diagnostic vaccination, Pneumococcal vaccines, Primary immunodeficiency, Polysaccharide responsiveness percentile

## Abstract

**Background:**

The assessment of polysaccharide responsiveness via vaccination is pivotal in the evaluation of patients for primary immunodeficiency. However, the applicability of current guidelines provided by the American Academy of Allergy, Asthma & Immunology (AAAAI) has been subject to scrutiny.

**Methods:**

We conducted a prospective study involving 120 healthy Danish adult blood donors. Antibodies targeting pneumococcal capsular polysaccharide serotypes were quantified using a multianalyte bead immunoassay before and four to eight weeks post-vaccination. Polysaccharide responsiveness in donors was assessed according to AAAAI guidelines.

**Results:**

Remarkably, only a minority of participants (2.5%) demonstrated a normal polysaccharide response per AAAAI criteria. This finding prompted us to advocate for an alternative approach based on percentile rankings relative to a reference population. Polysaccharide Responsiveness Percentile (PRP) was not significantly associated with age, sex, vaccine batch, or the duration between vaccination and antibody measurements in our cohort supporting its robustness, generalizability, and potential for standardized clinical application.

**Conclusion:**

Our study unveils significant limitations of the AAAAI guidelines, highlighting the imperative for a more robust and adaptable approach. By introducing a novel PRP assessment method, we aim to enhance the accuracy and reliability of immune function evaluations.

**Supplementary Information:**

The online version contains supplementary material available at 10.1007/s10875-025-01915-w.

## Introduction

Evaluating polysaccharide responsiveness—the ability to mount an antibody response to polysaccharide vaccines—plays a pivotal role in diagnosing primary immunodeficiencies [1, 2]. Deficient polysaccharide responsiveness is considered among the most common findings in patients suspected of having primary immunodeficiency diseases [3]. To standardize the assessment, the American Academy of Allergy, Asthma & Immunology (AAAAI) has established guidelines recommending measurement of antibodies against multiple pneumococcal capsular polysaccharide (CPS) serotypes before and four to eight weeks after immunization with the 23-valent pneumococcal polysaccharide vaccine (PPV), though the guidelines do not specify the exact number of antibodies or which serotype-specific antibodies should be assessed [4].

Under these guidelines, polysaccharide responsiveness is evaluated based on the number of specific anti-CPS antibodies that reach or exceeds a concentration threshold of 1.3 mg/L post-vaccination. In adults, responses are categorized as follows:

- **Severe deficiency**: Fewer than three antibodies reach a concentration of 1.3 mg/L (though the total number of antibodies to be tested is not defined).
- **Moderate deficiency**: Less than 70% of the tested antibodies reach a concentration of 1.3 mg/L.
- **Mild deficiency**: Multiple antibodies do not reach a concentration of 1.3 mg/L, or fewer than 70% show a two-fold increase from pre-vaccination levels.

Despite their widespread use, these guidelines are based on expert consensus rather than data-driven models from individuals with or without functional antibody deficiencies [4, 5], and lack rigorous validation. Studies have suggested that a significant proportion of individuals without suspected antibody deficiency, ranging from 20 to 60%, meet the AAAAI criteria for moderate or severe deficiency [6, 7]. This potential for false-positive results raises concerns about misdiagnosed immunodeficiency, leading to unnecessary patient anxiety, treatment exposure, and healthcare resource strain, highlighting the need to assess whether current guidelines are overly sensitive and to explore alternative approaches.

The applicability of the AAAAI guidelines is further complicated by variability in testing methods, which can significantly affect outcomes in polysaccharide responsiveness evaluations. Firstly, results depend heavily on the specific antibodies selected for measurement, as certain antibodies are more likely to surpass the 1.3 mg/L threshold than others [6, 8, 9]. Selection alone can increase or decrease the likelihood of being categorized as deficient by as much as four-fold [6]. Secondly, the shift from enzyme-linked immunosorbent assay (ELISA)—the method used when the guidelines were formulated—to multianalyte bead-based assays raises further challenges. These multiplex assays, now standard in anti-CPS antibody measurement, might produce variable results across platforms. Despite efforts to standardize multiplex assays in comparison to traditional ELISA assays [10], the transition to these newer platforms continues to complicate direct comparisons with the original ELISA-based results [11–15]. Consequently, polysaccharide responsiveness criteria may need to be recalibrated to reflect this transition.

Additional issues stem from the shared 1.3 mg/L cutoff across diverse antibodies, which may lack both clinical and biological relevance. Interpreting antibody concentrations as continuous variables, rather than applying a dichotomous threshold, could yield a more accurate representation of individual immune responses. Finally, the guidelines’ lack of clear criteria regarding the number and selection of antibodies introduces complexity and potential bias, as the number of antibodies measured can influence assessment outcomes [6, 16]. These limitations further stress the need for refined approaches in interpreting PPV response outcomes.

In this study, we aimed to assess polysaccharide responsiveness using the AAAAI guidelines in a cohort of healthy blood donors to determine the false-positive rate. In this context, false positives refer to individuals who are incorrectly classified by the guidelines as having a polysaccharide antibody deficiency, despite being presumed immunocompetent based on their eligibility and status as healthy blood donors. Furthermore, we propose an alternative framework for evaluating patient polysaccharide responsiveness, using this healthy cohort as a reference population.

## Methods

### Participant Recruitment and Sample Collection

Participants were recruited from the Blood Bank at Aarhus University Hospital, Denmark. The inclusion criteria were: (i) compliance with requirements for blood donation according to current Danish legislation, (ii) active blood donor, (iii) aged at least 18 years, (iv) understanding of the written and verbal description of the project, and (v) provision of written consent for participation. The exclusion criteria were: (i) previous vaccination with any pneumococcal vaccine, (ii) previous allergic reaction after any vaccination, or (iii) pregnancy.

Participants were instructed to report potential adverse events beyond those listed as ‘common’ in the package insert.

A total of 120 participants provided a baseline blood sample, received a single dose of PPV (Pneumovax, Merck Sharp & Dohme B.V., Netherlands) in a deltoid muscle, and provided a follow-up blood sample 4 to 8 weeks later. An additional four blood donors did not provide the follow-up sample and were therefore excluded from the study.

The blood samples drawn were 9 mL of unstabilized, peripheral venous blood. The blood was allowed to coagulate at ambient temperature for at least 30 min. Samples were then subjected to centrifugation at 2049 g for 4 min. The serum was collected, aliquoted in vials of ~ 1 mL, and stored at −80 °C until analysis.

### Multianalyte Bead Immunoassay

Serum IgG anti-CPS antibody concentrations were determined for each of twelve CPS included in PPV (1, 3, 4, 5, 6B, 7 F, 9 V, 14, 18 C, 19 A, 19 F, and 23 F), using an in-house Luminex method based on the procedure described by Lal et al. [17]. This method allows for the simultaneous measurement of all 12 analytes in each sample.

In brief, CPS obtained from LGC Standards (American Type Culture Collection, Virginia, USA) or SSI Diagnostica (Hilleroed, Denmark) were conjugated to poly-l-lysine and then covalently bound to carboxylated microspheres (Luminex, TX, USA). Serum samples were incubated with the conjugated microspheres, followed by incubation with R-phycoerythrin-conjugated goat anti-human IgG antibodies (Jackson ImmunoResearch Laboratories, West Grove, PA, USA). Each sample was analyzed in duplicate, and pre- and post-vaccination samples from each participant were tested simultaneously.

The microspheres were read on a Bio-Plex 200 system (Bio-Rad, Hercules, CA, USA), with data acquisition performed using Bio-Plex Manager 6.0 (Bio-Rad). A standard serum containing anti-CPS antibodies, calibrated to the 007sp reference serum (WHO 1 st International Standard for Human Anti-pneumococcal capsule Reference Serum) from the National Institute for Biological Standards and Control (Blanche Lane, Ridge, Herts, UK) was used as a reference. No formal external quality assurance programs are currently available, however, the assay was previously shown to perform comparably with other platforms in a European multilaboratory comparison study [10]. IgG anti-CPS antibody concentrations were calculated from the standard curve of median fluorescent intensity against known IgG anti-CPS antibody concentrations in the standard serum and converted to mg/L.

The assay is accredited according to ISO 17,025 by the Danish Accreditation Fund (DANAK).

### Categorization of Participants Using the AAAAI Guidelines

In instances where the AAAAI guidelines do not provide explicit rules, we made the following methodological decisions:

For the AAAAI criterion of mild deficiency, the phrase “Failure to generate protective titers to multiple serotypes” was interpreted as having three or more antibodies below 1.3 mg/L post-vaccination.

The AAAAI guidelines allow participants to meet the criteria for more than one category, such as both ‘severe’ and ‘moderate’ deficiency (e.g., if none of the tested antibodies reach a concentration of 1.3 mg/L). To ensure clear and consistent categorization in our study, we assigned each participant to a single category based on a hierarchical approach. Participants were classified in the first category they met in the following sequence: ‘severe’, ‘moderate’, ‘mild’, and ‘normal’.

### Statistics

False-positive rate was calculated as the number of false positives divided by the sum of false positives and true negative. Effect sizes between groups were determined with 95% confidence intervals (95%CI) using Estimation Statistics [18]. Linear regression analyses and figures were made in GraphPad Prism 10.2.3 (GraphPad Software, CA, USA).

### Ethics

The study complied with the principles of the Declaration of Helsinki and was approved by the Ethical Committee of the Central Denmark Region (September 20, 2022; reference ID: 1-10-72-48-22). Additionally, the study was registered in the internal data list of the Central Denmark Region (reference ID: 1-16-02-431-22). All participants provided written informed consent.

## Results

### Study Population

**Fig. 1:**
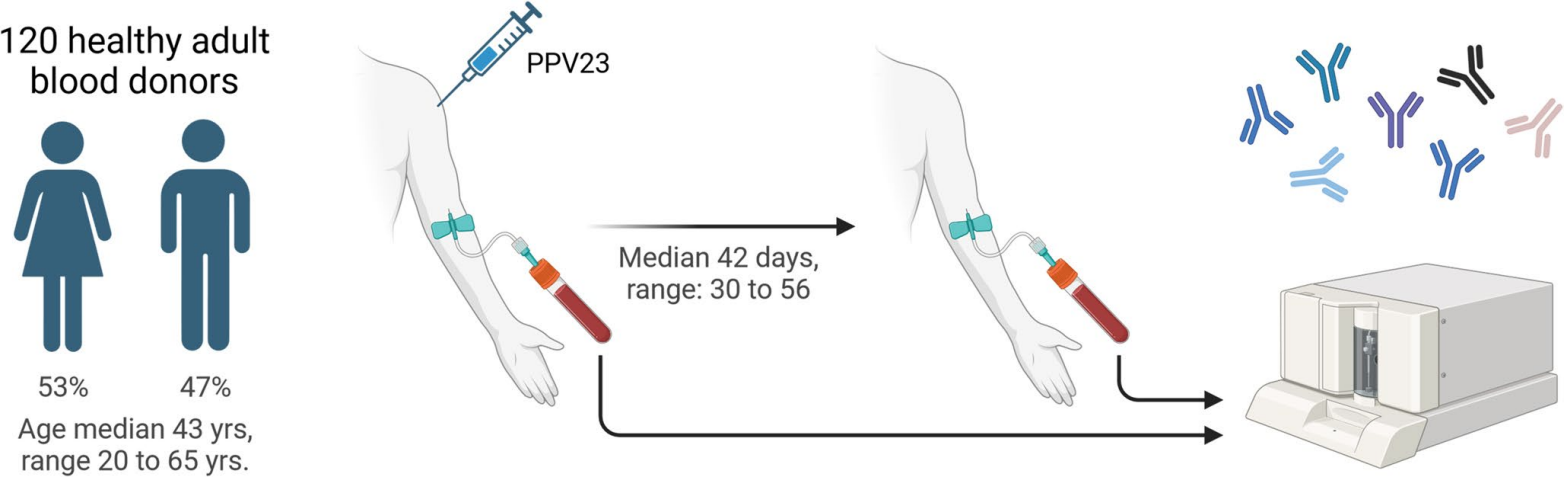
Overview of the Experimental Workflow: Blood samples were collected from participants, and the PPV vaccine was administered on the same day. A second blood sample was taken after a median of 42 days. All blood samples were analyzed in a single batch for IgG antibodies against 12 specific capsular polysaccharides (CPS) included in PPV using a multiplex, fluorescent bead-based assay

One hundred and twenty healthy adult Danish blood donors were recruited for the study (Fig. 1). The cohort included 64 females and 56 males. Participants’ ages had a median of 43 years, ranging from 20 to 65 years. Each participant provided a pre-vaccination blood sample, received the PPV vaccine, and then provided a post-vaccination blood sample. The pre-vaccination samples were collected on the day of vaccination. The median time from vaccination to post-vaccination sample collection was 42 days, with a range of 30 to 56 days. Pre- and post-vaccination samples were analyzed for IgG antibodies to twelve CPSs present in PPV (CPS 1, −3, −4, −5, −6B, −7 F, −9 V, −14, −18 C, −19 A, −19 F, and − 23 F), using a multianalyte bead immunoassay (Supplementary Table 1).

### Adverse Effects

No immediate hypersensitivity reactions to vaccination were observed. One potential adverse event was reported: symptoms of adhesive capsulitis of the shoulder, which gradually resolved with physiotherapy.

### Assessment of Polysaccharide Responsiveness in Danish Blood Donors using the AAAAI Guidelines

**Fig. 2:**
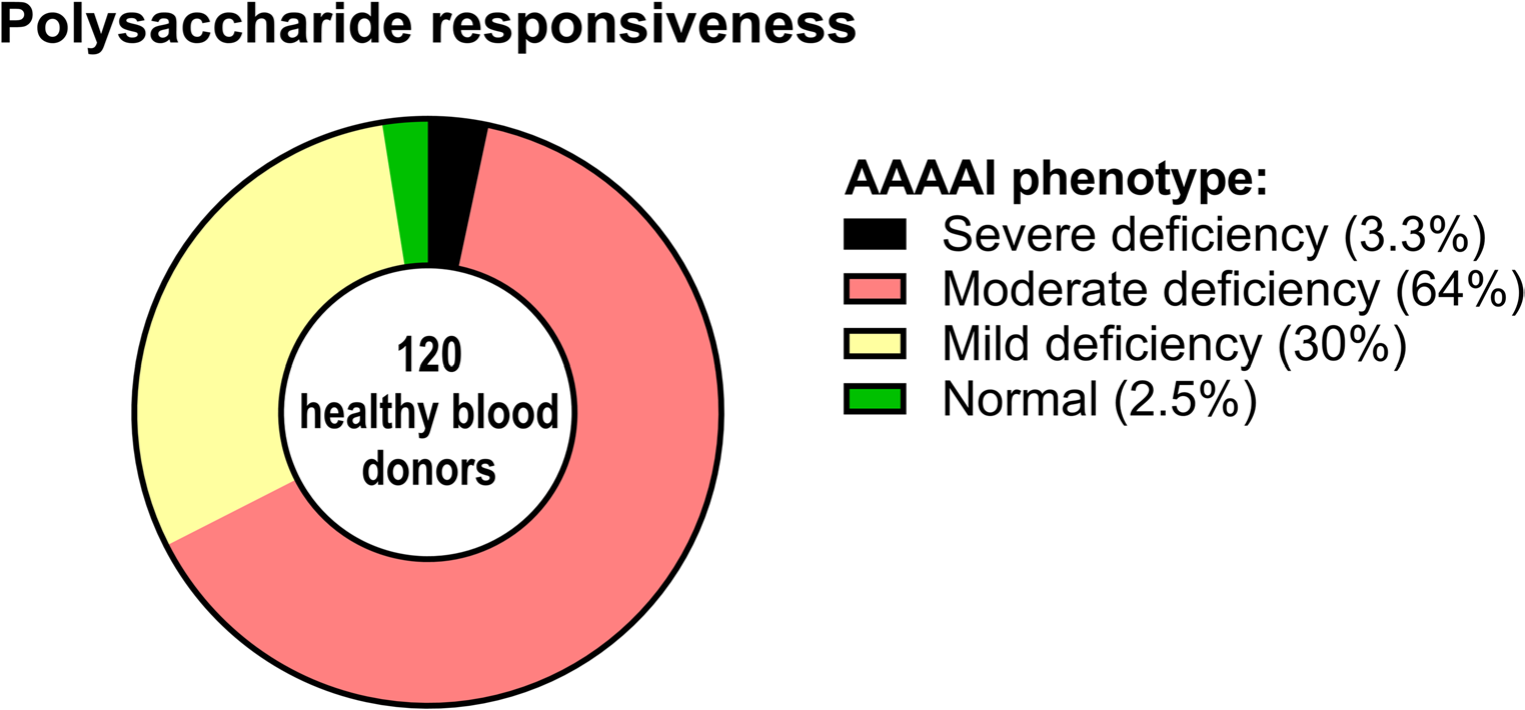
Assessment of Polysaccharide Responsiveness in Danish Blood Donors using the AAAAI Guidelines: Polysaccharide responsiveness was assessed following AAAAI guidelines. Antibody concentrations were measured in samples collected before and after vaccination. Interpretation criteria are as follows: Severe deficiency: Two or fewer antibodies with concentrations of 1.3 mg/L or higher after vaccination. Moderate deficiency: Fewer than 70% of antibodies with concentrations of 1.3 mg/L or higher after vaccination (corresponding maximum 8 out of 12 serotypes with concentrations of 1.3 mg/L or higher after vaccination in this specific assay). Mild deficiency: More than two antibodies with concentrations below 1.3 mg/L after vaccination or fewer than 70% of antibodies showing a two-fold or higher increase in concentration after vaccination compared to pre-vaccination levels. Normal response: Any response not meeting the criteria for severe, moderate, or mild deficiency. Participants were classified into a single category, representing the most severe deficiency level applicable

We applied the measured anti-CPS antibody concentrations to determine the participants’ polysaccharide responsiveness using the interpretation rules in the AAAAI guidelines [4]. The participants were Danish blood donors, who are generally healthier than the general population [19, 20] because individuals with recent illness, high-risk behaviors, chronic diseases, on medication etc. are excluded from donation. Strikingly, only three participants (2.5%) were classified as having a normal polysaccharide response (Fig. 2). Thirty-six participants (30%) had mild deficiency, 77 participants (64%) had moderate deficiency, and four participants (3.3%) had severe deficiency.

Under the assumption that none of the participants are truly polysaccharide responsiveness deficient, the false-positive rate for use of the AAAAI guidelines in this population is 97.5% (117/120). For moderate or severe deficiency, the false-positive rate is 67.5% (81/120).

Thus, even super-healthy individuals may have only minimal probabilities of classifying as having normal polysaccharide responsiveness according to the AAAAI guidelines. Consequently, we conclude that the guidelines are not useful for testing patients in our setting, and an alternative approach is therefore imperative.

### Antibody Concentrations Pre- and Post-Vaccination

As an alternative approach, we advocate expressing each patient’s response relative to a historical reference population. The participants in this study may serve as such a reference population. Previously, we proposed using Z-scores for this purpose, which required transforming measured antibody concentrations to approximate Gaussian distributions [6, 16].

**Fig. 3:**
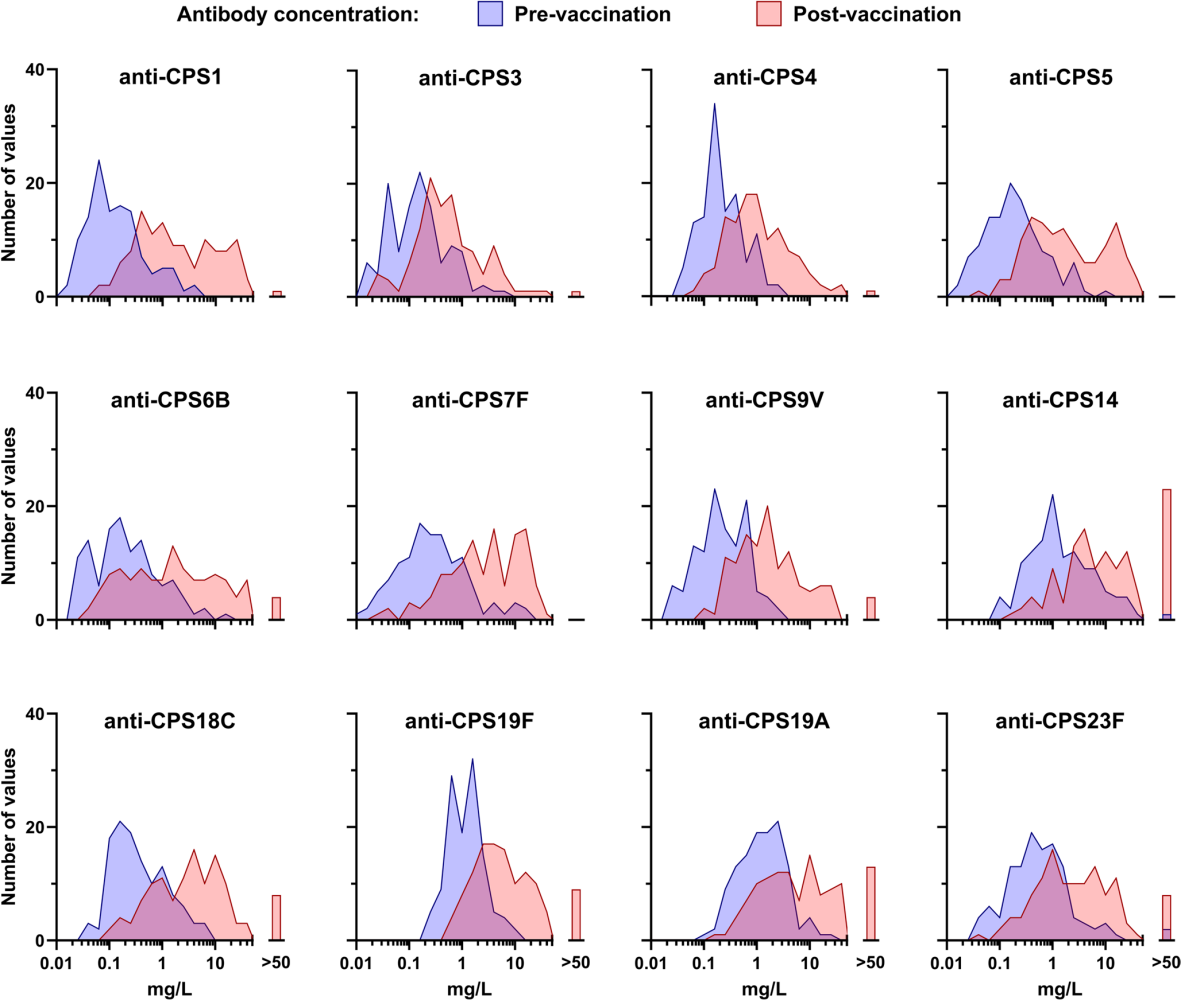
Antibody Concentrations Pre- and Post-Vaccination: Histogram representation of concentrations for each of the 12 antibodies measured in study participants. Concentrations measured in pre-vaccination samples are depicted in blue, while concentrations measured in post-vaccination samples are shown in red. Each bin represents a range of antibody concentrations with a width of 0.2 units on a logarithmic scale. The centers of neighboring bins are connected by straight lines. Antilog numbers are presented on the X-axis

We examined the distribution of antibody measurements in the cohort and found that pre-vaccination measurements generally approximated log-Gaussian distributions for each of the 12 antibodies (Fig. 3 and Supplementary Fig. 1). However, post-vaccination measurements only approximated such distributions for some antibodies, while others showed signs of dichotomous distributions (e.g., anti-CPS1 and anti-CPS5 antibodies). Additionally, for some antibodies, significant proportions of measurements exceeded the quantitative range of the assay used (e.g., for anti-CPS14 antibodies, 19% of measurements were above 50 mg/L).

Thus, an alternative approach to the Z-scores is needed to effectively utilize data from this cohort.

### The Polysaccharide Responsiveness Percentile (PRP)

**Fig. 4:**
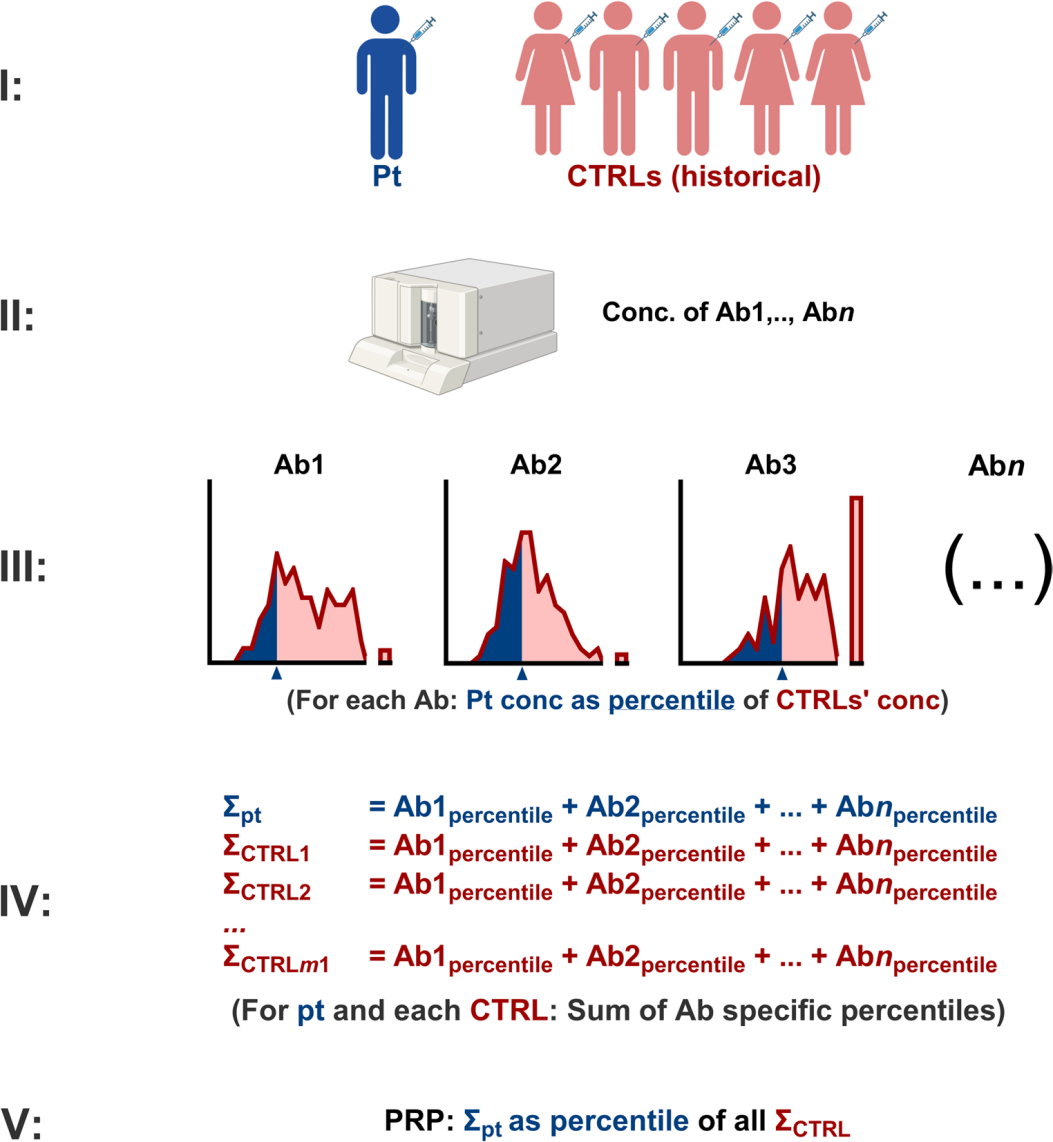
The Polysaccharide Responsiveness Percentile (PRP): Overview of the principles for calculating the Polysaccharide Responsiveness Percentile (PRP) for individual patients

To assess an individual patient’s polysaccharide responsiveness relative to a PPV-vaccinated reference population, we devised a simple, non-parametric approach, as illustrated in Fig. 4.

The proposed protocol consists of the following steps:

I. The patient receives a PPV vaccination.
II. Multiple anti-CPS antibodies are measured post-vaccination.
III. Each of the patient’s anti-CPS antibody measurements is transformed into the percentile of measurements for that specific antibody in the reference population.
IV. The patient’s percentiles for the different antibodies are summed.
V. The sum of the patient’s antibody percentiles is then transformed into the percentile of similar summed antibody percentiles in the reference population.

This final parameter is referred to as the Polysaccharide Responsiveness Percentile (PRP).

To ensure reliable comparison, the time elapsed after vaccination and the specific antibodies measured should be consistent between the patient and the reference population.

### Impact of Increasing Antibody Number on PRP Robustness

**Fig. 5:**
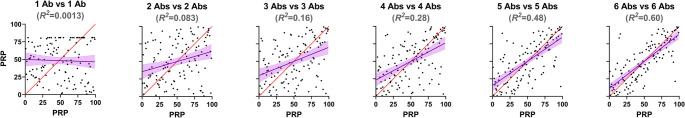
Impact of Increasing Antibody Number on PRP Robustness: Scatter plots illustrating the correlation between PRP of antibody sets, incorporating data from one to six different antibodies for individual participants. The antibody selection process aimed to generate sets with the weakest possible mutual correlation, as detailed in the supplementary data file. Linear regression curves with their 95% confidence intervals are depicted in purple, while the red lines represent the line of identity (y = x). Notably, as more antibodies are included in each set, the regression curve approximates the line of identity, and the R² value increases

Inclusion of more antibodies for calculating the PRP likely increases the robustness of results by reducing the impact of outliers. We examined the practical impact using the participants’ post-vaccination data by analyzing the correlations between PRPs determined from sets of antibodies, with each set containing data from between one and six antibodies. For antibody selection in each set, we applied a conservative approach, compiling data from antibodies that showed the weakest correlations based on their mutual Pearson’s correlation coefficients (Supplementary Fig. 2). Even with this conservative approach, we found that increasing the number of antibodies gradually increased the correlation between PRPs determined from different antibodies (Fig. 5).

We conclude that as many antibodies as possible should ideally be included in the PRP calculation. However, since the reduction of standard error is approximately inversely proportional to the square root of the antibody number (acknowledging that measurements from different antibodies in the same individual are not strictly independent), the marginal benefit of including more than 10–12 antibodies is subtle. Thus, including 10–12 antibodies may provide a pragmatic balance between robustness, practicality, and costs.

### PRP Variability Across Participant Characteristics

Several factors might impact PRP, including age, sex, vaccine batch, and the time elapsed from vaccination to post-vaccination anti-CPS antibody measurement. Given the potential variability of these factors among patients, we conducted a comprehensive analysis to assess their effects within our cohort, incorporating data for all twelve antibodies.

**Fig. 6:**
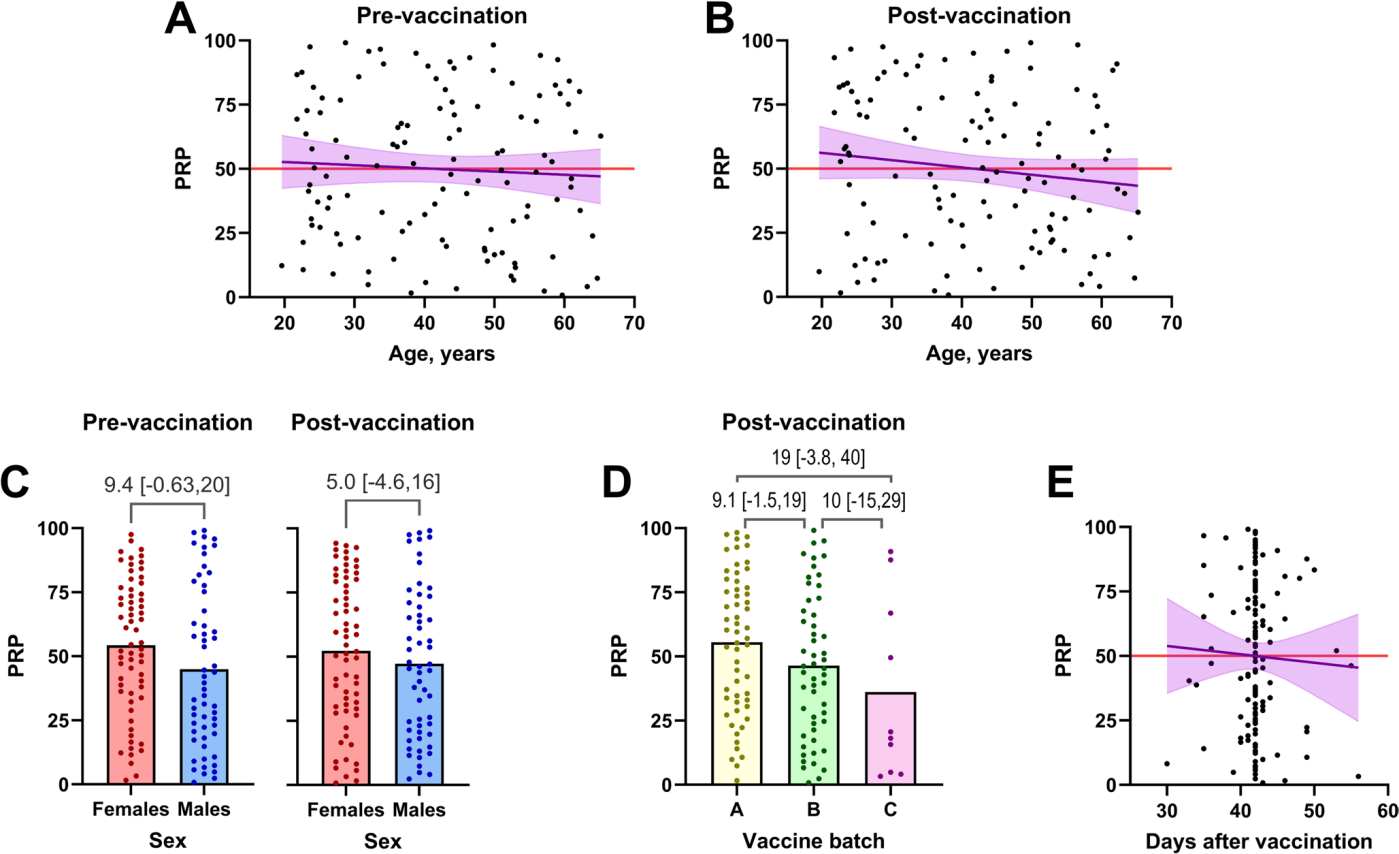
PRP Variability Across Participant Characteristics: **A**: Scatter plot showing the relationship between age and PRP incorporating all 12 measured antibodies pre-vaccination. Linear regression line with 95% confidence intervals is shown in purple. The red line represents y = 50, which falls within the confidence intervals of the regression line, indicating that the analysis does not support a significant relationship between age and the antibody measurements before vaccination. **B**: Scatter plot as described in panel A, but for PRP post-vaccination. The red line (y = 50) similarly falls within the confidence intervals of the regression line, indicating that the analysis does not support a significant relationship between age and antibody measurements after vaccination. **C**: Scatter plot depicting PRP pre-vaccination (left) and post-vaccination (right) vaccination, stratified by sex. Unpaired mean differences in PRP between sexes, with 95%CI, were calculated from 5000 bootstrap samples and are shown in grey. No significant differences were identified between the sexes at either time point. **D**: Scatter plot presenting post-vaccination PRP, stratified by vaccine batch (I: U019976, II: U023456, and III: U035558). Unpaired mean differences in PRP between batches, with 95%CI, were estimated similarly to panel C. No significant differences were found between vaccine batches. **E**: Scatter plot illustrating the relationship between post-vaccination PRP, and time elapsed from vaccination. Data analysis was performed as described for panel A. No significant relationship was observed between time from vaccination until determination of post-vaccination PRP

Our analysis found no statistically significant association between age and either pre-vaccination or post-vaccination PRP in a linear regression model (Fig. 6A and **B**). However, there was a slight trend towards decreasing post-vaccination PRPs with increasing age, with a point estimate of −0.28 percentiles per year (95%CI: −0.68, 0.11), i.e., not statistically different from 0.

Similarly, we observed no significant association between sex and PRP, both pre-vaccination and post-vaccination, although point estimates were slightly higher for females: 9.4 (95%CI: −0.63, 20) pre-vaccination and 5.0 (95%CI: −4.6, 16) post-vaccination (Fig. 6C).

Additionally, there was no significant association between vaccine batch and PRP (see Fig. 6D). Furthermore, the time elapsed from vaccination to post-vaccination anti-CPS antibody measurement showed no association with PRP (Fig. 6E). As a sensitivity analysis, participants with post-vaccination samples collected 42 days after vaccination (*n* = 62) were excluded, and PRPs were compared between participants with earlier and later collected post-vaccination samples. The unpaired mean difference in PRP was 1.53 (95%CI: −12.9, 16.0), i.e., not statistically significantly different from 0.

In summary, our analysis did not reveal any significant influence of these factors on PRP within our cohort. Although subtle effects cannot be entirely ruled out, they are likely negligible and can be disregarded when assessing individual adult patients using an adult reference population.

### Relationship Between Pre- and Post-Vaccination PRP

**Fig. 7:**
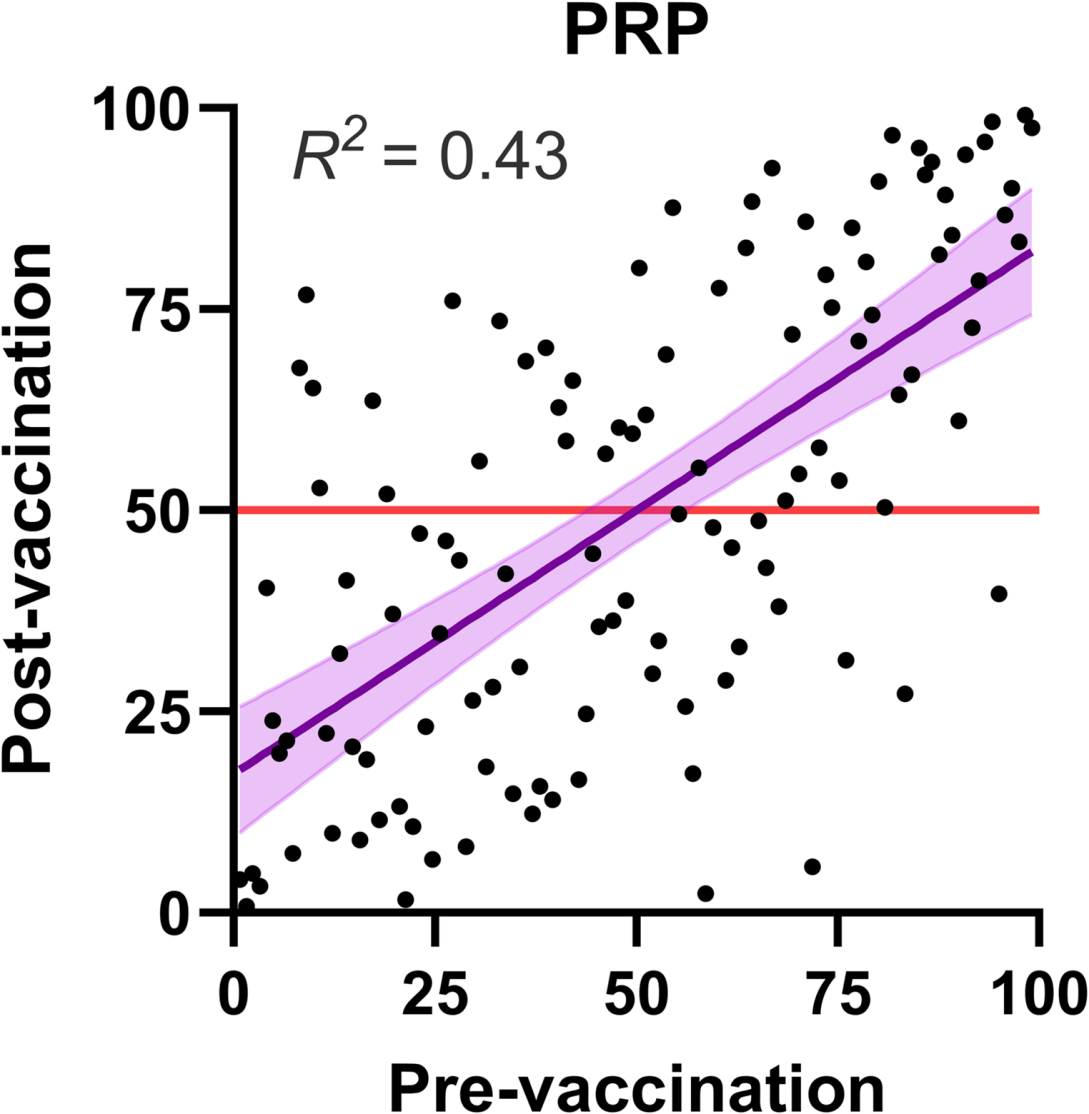
Relationship Between Pre- and Post-Vaccination PRP: Scatter plot showing the relationship between pre-vaccination PRP and post-vaccination PRP in the participants. The linear regression line with 95%CI is shown in purple. The red line represents *y* = 50, which does not fall within the confidence intervals of the regression line. This analysis supports a significant relationship between PRP measured before and after vaccination. Regression line slope estimate: 0.66 (95%CI: 0.52, 0.79)

We examined the correlation between pre-vaccination and post-vaccination PRP in our cohort, incorporating data for all twelve antibodies. Using a linear regression model, we observed a moderate to strong correlation, with an R-squared value of 0.43 and a slope estimate of 0.66 (95%CI: 0.52, 0.79) (Fig. 7). These findings demonstrate a significant relationship between pre-vaccination and post-vaccination PRP within the cohort. Thus, the level of naturally occurring anti-pneumococcal antibodies partially predicts the capacity assessed by diagnostic vaccination.

## Discussion

Our study found that only a minority of healthy Danish blood donors (2.5%) demonstrated a normal polysaccharide response according to AAAAI guidelines, highlighting significant limitations in these criteria. This strikingly low percentage suggests that the AAAAI criteria may be overly stringent, leading to a high rate of false positives. We propose an alternative assessment method based on percentile rankings relative to a reference population, which we term the Polysaccharide Responsiveness Percentile (PRP).

Martins et al. recently reported that approximately 20% of self-proclaimed healthy individuals had moderate or severe deficient polysaccharide responsiveness per the AAAAI guidelines [7]. Although these findings further support the notion that guidelines are overly sensitive, this proportion is notably lower than the 67% observed in our study. We hypothesize that the discrepancy may partly result from differences in assay calibrations, as well as the number and specificity of the measured antibodies. Notably, even when identical samples are analyzed using different assays, concordance rates for individual anti-CPS antibodies can be as low as 80% when applying the 1.3 mg/L threshold [21]. Overall conclusions regarding polysaccharide responsiveness may be similarly influenced [9]. Such inter-assay differences highlight the need for caution in applying threshold concentrations established by one assay to others. It is crucial that reference intervals are generated using the same assay employed for patient evaluation.

Borgers et al. improved upon existing methods by using the fifth percentile from a healthy reference population as a cutoff [15]. However, their continued reliance on a dichotomous assessment for individual anti-CPS antibodies limits the nuanced interpretation of patient results. Treating these measurements as continuous variables would provide a more comprehensive understanding of immune function. Furthermore, applying a uniform cutoff, such as the fifth percentile across all antibodies, may not capture clinically relevant variability in responses.

In light of the limitations of the AAAAI guidelines, we propose the PRP method as a novel approach for assessing polysaccharide responsiveness using percentile rankings relative to a reference population. This two-step approach involves PPV vaccination followed by a single blood sample collected 4 to 8 weeks later, excluding pre-vaccination measurements required by the AAAAI guidelines. This streamlined method reduces costs and simplifies logistics, minimizing the risk of procedural failure. By transforming individual antibody measurements into percentiles and summing these to generate an overall PRP, this approach better accommodates the inherent variability in antibody responses than the dichotomous AAAAI criteria.

Additionally, a key advantage of the PRP method is its ability to account for serotype-specific differences in immunogenicity by evaluating each antibody concentration relative to a healthy reference population, thereby reducing bias from inherently low- or high-responding serotypes.

Importantly, the PRP method is based on post-vaccination antibody levels, which reflect overall polysaccharide responsiveness rather than vaccine-induced fold changes per se. The standardized exposure provided by vaccination serves to reveal insufficient responsiveness, regardless of whether protective antibody levels were achieved through prior natural exposure or immunization.

Our analysis revealed no statistically significant associations between PRP and age, sex, vaccine batch, or the time elapsed from vaccination to post-vaccination antibody measurement. This suggests that the PRP method is broadly applicable across diverse adult patient demographics and procedural variables.

Strengths of our study include robust sample size and the use of a comprehensive multianalyte bead immunoassay. However, several limitations should be acknowledged. All samples were quantified in a single laboratory using one technology, although this assay was validated, and measurements were referenced to the WHO international standard serum for such antibodies. Additionally, the timing of post-vaccination sampling may not reflect typical clinical scenarios, as most samples were collected exactly 42 days after vaccination. However, we do not anticipate significant effects, as we observed no indications that earlier or later post-vaccination samples yielded different PRP scores.

Future research should focus on the robustness of the PRP method across different laboratories and antibody quantification technologies, as well as the number, specificity, and diagnostic contribution (including potential weighting) of the included antibodies. Given the potential for inter-laboratory variation in multiplex assay performance, cross-site validation is essential to ensure generalizability. The method should also be tested in historical patient cohorts to define rational PRP cutoffs for polysaccharide responsiveness, paving the way for subsequent testing in prospective clinical studies. Although the PRP method is not yet available for routine clinical use, collaborative research efforts are ongoing to define its clinical utility. The present study of healthy adults may serve as a reference cohort for future investigations, which should include patients with suspected or confirmed antibody deficiency to establish evidence-based diagnostic criteria. Cross-laboratory validation and studies across diverse patient populations will be essential for broader implementation. We welcome collaboration with other research groups to support the continued validation and refinement of the PRP method.

In conclusion, our study highlights significant limitations in the current AAAAI guidelines for assessing polysaccharide responsiveness, which pose a considerable risk of false-positive outcomes in healthy individuals. The PRP method represents a promising alternative that warrants further investigation and potential integration into clinical practice for enhanced decision-making.

## Electronic Supplementary Material

Below is the link to the electronic supplementary material.

Supplementary Material 1

## Data Availability

Data is provided within the supplementary information files.

## References

[1] Bonilla FA, Khan DA, Ballas ZK, Chinen J, Frank MM, Hsu JT, et al. Practice parameter for the diagnosis and management of primary immunodeficiency. J Allergy Clin Immunol. 2015;136(5):1186–205.e1-78. 26371839 10.1016/j.jaci.2015.04.049

[2] Bousfiha A, Jeddane L, Picard C, Al-Herz W, Ailal F, Chatila T, et al. Human inborn errors of immunity: 2019 update of the IUIS phenotypical classification. J Clin Immunol. 2020;40(1):66–81. 32048120 10.1007/s10875-020-00758-x PMC7082388

[3] Javier FC 3rd, Moore CM, Sorensen RU. Distribution of primary immunodeficiency diseases diagnosed in a pediatric tertiary hospital. Annals Allergy Asthma Immunology: Official Publication Am Coll Allergy Asthma Immunol. 2000;84(1):25–30. 10.1016/S1081-1206(10)62736-6 10674561

[4] Orange JS, Ballow M, Stiehm ER, Ballas ZK, Chinen J, De La Morena M, et al. Use and interpretation of diagnostic vaccination in primary immunodeficiency: a working group report of the basic and clinical immunology interest section of the American academy of allergy, asthma & immunology. J Allergy Clin Immunol. 2012;130(3 Suppl):S1–24. 22935624 10.1016/j.jaci.2012.07.002

[5] Sorensen RU, Leiva LE, Javier FC 3rd, Sacerdote DM, Bradford N, Butler B, et al. Influence of age on the response to Streptococcus pneumoniae vaccine in patients with recurrent infections and normal Immunoglobulin concentrations. J Allergy Clin Immunol. 1998;102(2):215–21. 9723664 10.1016/s0091-6749(98)70089-2

[6] Hansen AT, Söderström A, Jørgensen CS, Larsen CS, Petersen MS, Jensen B. Diagnostic vaccination in clinical practice. Front Immunol. 2021;12:717873. 34659207 10.3389/fimmu.2021.717873 PMC8514775

[7] Martins TB, Hill HR, Peterson LK. Evaluating patient immunocompetence through antibody response to Pneumococcal polysaccharide vaccine using a newly developed 23 serotype multiplexed assay. Clin Immunol. 2024;265:110295. 38914359 10.1016/j.clim.2024.110295

[8] Park MA, Jenkins SM, Smith CY, Pyle RC, Sacco KA, Ryu E, et al. Pneumococcal serotype-specific cut-offs based on antibody responses to Pneumococcal polysaccharide vaccination in healthy adults. Vaccine. 2021;39(21):2850–6. 33896666 10.1016/j.vaccine.2021.04.015

[9] Daly TM, Pickering JW, Zhang X, Prince HE, Hill HR. Multilaboratory assessment of threshold versus fold-change algorithms for minimizing analytical variability in multiplexed Pneumococcal IgG measurements. Clin Vaccine Immunol. 2014;21(7):982–8. 24807051 10.1128/CVI.00235-14 PMC4097436

[10] Meek B, Ekström N, Kantsø B, Almond R, Findlow J, Gritzfeld JF et al. Multilaboratory comparison of Pneumococcal multiplex immunoassays used in immunosurveillance of Streptococcus pneumoniae across Europe. mSphere 2019;4(6). 10.1128/mSphere.00455-19 PMC6881716 31776237

[11] Marsh RA, Orange JS. Antibody deficiency testing for primary immunodeficiency: A practical review for the clinician. Ann Allergy Asthma Immunol. 2019;123(5):444–53. 31446132 10.1016/j.anai.2019.08.012

[12] Sorensen RU, Leiva LE. Measurement of Pneumococcal polysaccharide antibodies. J Clin Immunol. 2014;34(2):127–8. 24337649 10.1007/s10875-013-9977-z

[13] Balloch A, Licciardi PV, Tang ML. Serotype-specific anti-pneumococcal IgG and immune competence: critical differences in interpretation criteria when different methods are used. J Clin Immunol. 2013;33(2):335–41. 23054341 10.1007/s10875-012-9806-9

[14] Klein DL, Martinez JE, Hickey MH, Hassouna F, Zaman K, Steinhoff M. Development and characterization of a multiplex bead-based immunoassay to quantify Pneumococcal capsular polysaccharide-specific antibodies. Clin Vaccine Immunol. 2012;19(8):1276–82. 22739691 10.1128/CVI.05535-11 PMC3416098

[15] Borgers H, Moens L, Picard C, Jeurissen A, Raes M, Sauer K, et al. Laboratory diagnosis of specific antibody deficiency to Pneumococcal capsular polysaccharide antigens by multiplexed bead assay. Clin Immunol. 2010;134(2):198–205. 19914139 10.1016/j.clim.2009.10.006

[16] Bernth-Jensen JM, Søgaard OS. Polysaccharide responsiveness is not biased by prior pneumococcal-conjugate vaccination. PLoS ONE. 2013;8(10):e75944. 24146796 10.1371/journal.pone.0075944 PMC3795730

[17] Lal G, Balmer P, Stanford E, Martin S, Warrington R, Borrow R. Development and validation of a nonaplex assay for the simultaneous quantitation of antibodies to nine Streptococcus pneumoniae serotypes. J Immunol Methods. 2005;296(1–2):135–47. 15680158 10.1016/j.jim.2004.11.006

[18] Ho J, Tumkaya T, Aryal S, Choi H, Claridge-Chang A. Moving beyond P values: data analysis with Estimation graphics. Nat Methods. 2019;16(7):565–6. 31217592 10.1038/s41592-019-0470-3

[19] Ullum H, Rostgaard K, Kamper-Jørgensen M, Reilly M, Melbye M, Nyrén O, et al. Blood donation and blood donor mortality after adjustment for a healthy donor effect. Transfusion. 2015;55(10):2479–85. 26098293 10.1111/trf.13205

[20] Brodersen T, Rostgaard K, Lau CJ, Juel K, Erikstrup C, Nielsen KR, et al. The healthy donor effect and survey participation, becoming a donor and donor career. Transfusion. 2023;63(1):143–55. 36479702 10.1111/trf.17190 PMC10107247

[21] Zhang X, Simmerman K, Yen-Lieberman B, Daly TM. Impact of analytical variability on clinical interpretation of multiplex Pneumococcal serology assays. Clin Vaccine Immunol. 2013;20(7):957–61. 23677324 10.1128/CVI.00223-13 PMC3697459

