# Supplementary material for: Reassessing Polysaccharide Responsiveness: Unveiling Limitations of Current Guidelines and Introducing the Polysaccharide Responsiveness Percentile Approach
Source: J Clin Immunol. 2025 Jul 25;45(1):115. doi: 10.1007/s10875-025-01915-w (PMC12296993; doi:10.1007/s10875-025-01915-w)
Supplement: Supplementary file 1 — Supplementary Material 1 [file 10875_2025_1915_MOESM1_ESM.docx]

### Supplementary Table 1. Anti-CPS antibody measurements in participants.

|  | **Antibody against Capsular Polysaccharide Serotype (mg/L):** | | | | | | | | |  |  |  |
| --- | --- | --- | --- | --- | --- | --- | --- | --- | --- | --- | --- | --- |
| **Sample** | **1** | **3** | **4** | **5** | **6B** | **7F** | **9V** | **14** | **18C** | **19A** | **19F** | **23F** |
| D001_pre | 0.12 | 0.21 | 0.24 | 0.31 | 0.14 | 0.45 | 0.60 | 0.88 | 0.16 | 2.26 | 1.21 | >50 |
| D001_post | 0.14 | 0.32 | 0.83 | 0.77 | 0.18 | 11.85 | 4.52 | 8.50 | 0.40 | 23.77 | 8.46 | >50 |
| D002_pre | 0.14 | 0.25 | 0.36 | 0.34 | 0.18 | 1.10 | 0.65 | 2.45 | 0.63 | 1.58 | 1.56 | 0.34 |
| D002_post | 1.92 | 38.49 | 0.77 | 27.80 | 1.45 | 22.52 | 0.60 | >50 | >50 | 27.87 | 6.31 | 0.82 |
| D003_pre | 0.08 | 0.17 | 0.18 | 0.97 | 0.24 | 0.07 | 0.09 | 0.62 | 0.14 | 1.15 | 0.78 | 0.39 |
| D003_post | 0.52 | 0.09 | 0.12 | 32.86 | 0.32 | 0.13 | 0.32 | 5.01 | 0.20 | 3.67 | 19.66 | 0.36 |
| D004_pre | 0.08 | 0.42 | 0.33 | 0.08 | 0.05 | 0.59 | 1.25 | 0.15 | 0.10 | 0.84 | 0.48 | 0.94 |
| D004_post | 1.81 | 0.30 | 1.29 | 1.28 | 0.27 | 14.26 | >50 | 0.27 | 3.19 | >50 | 17.70 | 6.02 |
| D005_pre | 0.03 | 0.02 | 0.06 | 0.03 | 0.12 | 0.83 | 0.03 | 4.10 | 0.04 | 2.12 | 0.51 | 1.85 |
| D005_post | 0.87 | 0.02 | 0.11 | 0.72 | 0.22 | 4.70 | 0.08 | 10.82 | 0.25 | 2.53 | 1.46 | 7.86 |
| D006_pre | 0.07 | 0.07 | 0.69 | 2.84 | 0.26 | 0.26 | 2.79 | 5.04 | 0.13 | 0.46 | 2.67 | 0.21 |
| D006_post | 7.99 | 4.21 | 6.91 | 34.22 | 6.52 | 11.58 | >50 | >50 | 0.95 | 1.38 | >50 | 0.62 |
| D007_pre | 0.26 | 0.25 | 0.65 | 0.12 | 0.73 | 0.33 | 0.55 | 0.97 | 0.80 | 1.31 | 1.48 | 0.76 |
| D007_post | 11.37 | 0.22 | 3.54 | 1.07 | 1.47 | 0.32 | 1.40 | 2.20 | 4.00 | 1.07 | 3.11 | 0.88 |
| D008_pre | 0.67 | 0.20 | 0.40 | 0.56 | 0.36 | 17.91 | 0.67 | 31.71 | 3.88 | 1.39 | 2.71 | 1.12 |
| D008_post | 0.78 | 0.19 | 1.56 | 0.61 | 0.41 | 17.14 | 1.18 | 29.28 | 3.46 | 1.48 | 3.25 | 1.22 |
| D009_pre | 0.11 | 0.14 | 0.32 | 0.18 | 0.13 | 0.06 | 0.59 | 6.15 | 1.07 | 1.60 | 1.70 | >50 |
| D009_post | 0.90 | 1.96 | 12.41 | 0.37 | 1.03 | 0.19 | 1.94 | 14.49 | 6.62 | 6.43 | 2.82 | >50 |
| D010_pre | 0.14 | 0.03 | 0.07 | 0.06 | 0.17 | 0.14 | 0.10 | 0.71 | 0.33 | 3.62 | 0.48 | 0.47 |
| D010_post | 7.25 | 0.77 | 0.32 | 0.46 | 2.87 | 2.19 | 0.64 | 29.57 | 2.47 | 9.36 | 4.07 | 1.18 |
| D011_pre | 0.04 | 0.19 | 0.11 | 0.54 | 0.03 | 0.02 | 0.06 | 1.56 | 0.48 | 0.69 | 0.46 | 0.17 |
| D011_post | 0.80 | 0.22 | 0.79 | 28.45 | 0.10 | 0.35 | 0.78 | >50 | >50 | 9.01 | >50 | 0.73 |
| D012_pre | 0.05 | 0.03 | 0.20 | 0.51 | 0.04 | 0.16 | 0.09 | 1.81 | 0.15 | 0.60 | 1.70 | 0.26 |
| D012_post | 0.27 | 0.29 | 0.37 | 3.55 | 0.12 | 1.09 | 0.24 | 21.31 | 0.16 | 0.49 | 2.46 | 0.93 |
| D013_pre | 0.43 | 0.01 | 0.04 | 0.07 | 0.25 | 0.10 | 0.06 | 5.92 | 0.05 | 0.95 | 0.76 | 0.09 |
| D013_post | 41.51 | 0.14 | 0.72 | 13.87 | 4.19 | 0.56 | 6.41 | >50 | 0.46 | >50 | 28.41 | 0.34 |
| D014_pre | 0.23 | 0.21 | 0.20 | 0.96 | 0.10 | 2.72 | 0.79 | 3.43 | 0.28 | 1.08 | 1.22 | 1.08 |
| D014_post | 0.93 | 3.29 | 2.07 | 1.83 | 1.47 | 21.61 | 3.08 | >50 | 4.58 | 2.74 | 25.82 | >50 |
| D015_pre | 0.33 | 0.04 | 0.16 | 0.10 | 0.13 | 0.08 | 0.11 | 0.39 | 0.13 | 0.79 | 0.78 | 0.26 |
| D015_post | 33.09 | 0.18 | 0.23 | 1.07 | 1.26 | 0.49 | 0.39 | 0.93 | 0.81 | 1.70 | 1.83 | 1.62 |
| D016_pre | 0.05 | 1.23 | 0.10 | 0.04 | 0.05 | 0.02 | 0.14 | 5.46 | 0.08 | 0.64 | 1.10 | 0.12 |
| D016_post | 0.07 | >50 | 0.13 | 0.96 | 0.10 | 0.05 | 0.43 | 16.26 | 0.10 | 4.64 | 3.07 | 0.17 |
| D017_pre | 0.15 | 0.23 | 0.43 | 8.21 | 6.12 | 1.19 | 0.66 | 1.43 | 0.59 | 6.68 | 1.41 | 7.73 |
| D017_post | 7.59 | 0.45 | >50 | 32.16 | >50 | 12.89 | 14.06 | 3.92 | 18.14 | 10.71 | 7.22 | 13.45 |
| D018_pre | 0.08 | 0.10 | 0.25 | 0.16 | 2.79 | 0.17 | 0.41 | 0.78 | 1.32 | 0.81 | 0.92 | 0.37 |
| D018_post | 0.36 | 0.35 | 1.44 | 2.47 | 4.54 | 1.28 | 3.19 | 0.99 | 6.28 | 1.37 | 1.78 | 0.32 |
| D019_pre | 0.29 | 0.60 | 1.70 | 0.40 | 0.79 | 0.31 | 0.87 | 24.48 | 5.52 | 2.91 | 4.93 | 2.40 |
| D019_post | 7.35 | 4.78 | 3.09 | 6.65 | 1.31 | 11.60 | 4.44 | >50 | >50 | 2.56 | 8.50 | 6.75 |
| D020_pre | 0.24 | 0.26 | 0.81 | 0.27 | 0.94 | 1.46 | 1.36 | 1.43 | 2.11 | 1.98 | 3.69 | 4.11 |
| D020_post | 0.91 | 0.30 | 3.76 | 0.53 | 8.15 | 15.76 | 25.72 | 3.55 | 28.36 | 2.38 | 13.59 | >50 |
| D021_pre | 0.08 | 0.24 | 0.21 | 0.18 | 0.10 | 0.08 | 0.60 | 6.81 | 2.76 | 2.62 | 1.56 | 1.15 |
| D021_post | 0.16 | 0.22 | 0.12 | 0.34 | 0.28 | 0.12 | 0.28 | 8.68 | 12.78 | 2.70 | 1.35 | 0.94 |
| D022_pre | 0.08 | 0.13 | 0.24 | 0.58 | 0.39 | 10.29 | 0.36 | 1.07 | 0.54 | 2.49 | 9.43 | 0.73 |
| D022_post | 2.79 | 0.33 | 0.24 | 1.72 | 3.13 | 8.38 | 0.59 | 5.17 | 2.22 | 4.87 | 32.37 | 1.41 |
| D023_pre | 0.07 | 0.19 | 0.18 | 0.17 | 0.37 | 0.25 | 0.39 | 1.86 | 0.42 | 2.06 | 1.86 | 1.38 |
| D023_post | 3.15 | 0.85 | 0.59 | 5.62 | 14.12 | 1.30 | 1.27 | 2.36 | 4.88 | 13.19 | 20.66 | 18.61 |
| D024_pre | 0.07 | 0.11 | 0.18 | 0.07 | 0.10 | 0.15 | 0.29 | 0.96 | 0.12 | 0.86 | 0.80 | 0.70 |
| D024_post | 2.92 | 0.62 | 2.11 | 0.33 | 0.93 | 1.63 | 2.11 | 14.92 | 0.78 | 7.33 | 3.34 | 1.27 |
| D025_pre | 0.28 | 0.45 | 0.34 | 2.75 | 2.93 | 9.21 | 1.17 | 5.89 | 4.07 | 2.19 | 2.35 | 3.15 |
| D025_post | 13.29 | 2.70 | 6.67 | 17.89 | 45.26 | 16.19 | 20.07 | >50 | >50 | 43.77 | 46.65 | 15.29 |
| D026_pre | 0.06 | 0.32 | 0.14 | 0.26 | 0.17 | 8.56 | 0.09 | 0.54 | 1.57 | 0.87 | 1.73 | 1.90 |
| D026_post | 1.20 | 0.77 | 0.33 | 17.19 | 3.38 | 15.97 | 2.09 | 20.30 | 25.08 | 0.89 | 6.95 | >50 |
| D027_pre | 0.08 | 0.03 | 0.30 | 0.23 | 0.12 | 0.09 | 0.69 | 0.27 | 4.93 | 0.88 | 3.08 | 0.59 |
| D027_post | 5.26 | 0.26 | 4.28 | 4.46 | 0.76 | 0.28 | 3.83 | 0.70 | >50 | 17.58 | 19.88 | 1.30 |
| D028_pre | 0.27 | 0.83 | 0.24 | 0.08 | 13.17 | 0.08 | 0.40 | 0.80 | 0.19 | 0.99 | 1.14 | 1.74 |
| D028_post | 0.56 | 0.67 | 2.49 | 0.17 | 18.60 | 0.95 | 19.12 | 24.03 | 0.45 | 1.29 | 2.51 | 3.38 |
| D029_pre | 0.08 | 0.14 | 0.12 | 0.15 | 1.28 | 1.71 | 0.13 | 1.07 | 5.27 | 0.96 | 1.12 | 19.51 |
| D029_post | 0.41 | 0.11 | 0.18 | 0.44 | 16.49 | 11.32 | 0.41 | 22.96 | >50 | 1.53 | 5.90 | >50 |
| D030_pre | 0.25 | 0.13 | 0.10 | 1.14 | 0.70 | 0.03 | 0.41 | 1.41 | 0.16 | 0.40 | 0.50 | 0.29 |
| D030_post | 3.27 | 0.19 | 2.79 | 11.60 | >50 | 0.27 | 24.59 | 2.13 | 4.17 | 1.53 | 5.87 | 4.55 |
| D031_pre | 0.06 | 0.17 | 0.14 | 0.31 | 0.04 | 0.05 | 0.43 | 1.03 | 0.41 | 1.79 | 0.73 | 0.84 |
| D031_post | 0.33 | 0.79 | 0.26 | 8.36 | 0.07 | 0.82 | 0.83 | 2.75 | 6.61 | 23.47 | 1.16 | 1.15 |
| D032_pre | 0.06 | 0.02 | 0.18 | 0.09 | 0.04 | 0.02 | 0.15 | 20.64 | 0.08 | 0.63 | 1.74 | 0.84 |
| D032_post | 0.86 | 0.04 | 0.30 | 0.43 | 0.12 | 0.05 | 0.37 | 28.69 | 0.22 | 2.80 | 2.40 | 2.42 |
| D033_pre | 0.05 | 0.04 | 0.17 | 0.30 | 0.09 | 0.34 | 0.08 | 0.40 | 0.12 | 1.42 | 0.61 | 0.15 |
| D033_post | 0.15 | 0.64 | 0.42 | 8.02 | 0.14 | 4.93 | 0.79 | 3.72 | 0.49 | 33.74 | 1.03 | 0.25 |
| D034_pre | 0.03 | 0.09 | 0.24 | 0.24 | 0.12 | 14.56 | 0.21 | 0.47 | 0.11 | 1.16 | 0.59 | 0.20 |
| D034_post | 0.07 | 0.61 | 1.64 | 0.63 | 0.74 | 16.00 | 1.57 | 3.36 | 0.51 | 4.30 | 2.57 | 0.87 |
| D035_pre | 0.03 | 0.16 | 0.17 | 0.03 | 0.29 | 0.59 | 0.10 | 0.08 | 0.39 | 1.17 | 1.53 | 0.14 |
| D035_post | 4.57 | 0.66 | 0.63 | 0.14 | 0.82 | 10.90 | 1.81 | 6.75 | 1.35 | 12.01 | >50 | 2.41 |
| D036_pre | 0.14 | 5.59 | 0.19 | 0.10 | 0.74 | 1.23 | 0.12 | 0.69 | 0.38 | 1.48 | 0.90 | 0.20 |
| D036_post | 0.35 | 6.12 | 0.24 | 0.23 | 4.55 | 2.06 | 0.30 | 3.89 | 0.68 | 1.52 | 1.15 | 6.52 |
| D037_pre | 0.27 | 1.00 | 0.35 | 0.20 | 0.15 | 0.36 | 0.17 | 0.73 | 0.96 | 1.78 | 1.28 | 0.40 |
| D037_post | 5.63 | 1.13 | 0.57 | 2.18 | 0.16 | 1.94 | 1.49 | 2.00 | 3.50 | 5.09 | 4.60 | 0.57 |
| D038_pre | 0.65 | 2.56 | 2.33 | 0.90 | 0.11 | 0.54 | 1.86 | 17.33 | 0.24 | 1.59 | 3.10 | 9.27 |
| D038_post | 14.38 | 1.91 | 1.94 | 2.39 | 0.45 | 0.84 | 1.79 | >50 | 2.22 | 3.03 | 15.79 | 11.66 |
| D039_pre | 0.42 | 0.04 | 0.14 | 0.58 | 0.07 | 0.06 | 0.13 | 0.62 | 0.16 | 0.64 | 1.62 | 0.42 |
| D039_post | 6.96 | 2.14 | 0.30 | 2.60 | 0.07 | 0.88 | 0.30 | 0.82 | 0.18 | 3.56 | 3.44 | 0.40 |
| D040_pre | 1.13 | 0.21 | 0.29 | 0.13 | 1.00 | 0.69 | 0.44 | 7.70 | 1.13 | 3.44 | 2.33 | 1.87 |
| D040_post | 1.34 | 0.32 | 1.07 | 9.17 | 9.90 | 6.84 | 4.38 | >50 | >50 | 41.68 | 7.07 | 18.95 |
| D041_pre | 0.06 | 0.04 | 0.08 | 0.03 | 0.03 | 0.08 | 0.03 | 0.23 | 2.58 | 0.44 | 1.63 | 0.19 |
| D041_post | 0.42 | 0.36 | 6.96 | 0.20 | 1.38 | 1.90 | 1.38 | 45.36 | >50 | >50 | 38.16 | 1.28 |
| D042_pre | 0.05 | 0.16 | 0.48 | 0.12 | 1.04 | 0.05 | 0.15 | 0.42 | 0.10 | 0.34 | 0.42 | 0.42 |
| D042_post | 0.54 | 4.15 | 8.39 | 11.85 | 2.88 | 0.59 | 1.92 | 3.07 | 0.46 | 0.62 | 0.76 | >50 |
| D043_pre | 0.92 | 0.26 | 0.27 | 0.13 | 0.20 | 0.30 | 0.19 | 0.24 | 0.72 | 3.68 | 0.87 | 0.26 |
| D043_post | 1.77 | 0.23 | 0.34 | 0.22 | 0.43 | 0.40 | 0.26 | 0.24 | 1.11 | 3.79 | 0.83 | 0.42 |
| D044_pre | 0.08 | 0.05 | 0.14 | 0.22 | 0.30 | 0.41 | 0.26 | 0.83 | 0.39 | 0.71 | 0.70 | 1.36 |
| D044_post | 0.24 | 0.60 | 0.35 | 1.02 | 7.07 | 1.46 | 1.22 | 3.85 | 8.53 | 3.19 | 4.42 | 3.67 |
| D045_pre | 0.30 | 1.25 | 1.84 | 0.16 | 0.79 | 1.04 | 0.53 | 2.37 | 0.87 | 1.46 | 2.17 | 1.00 |
| D045_post | 2.71 | 1.48 | 27.21 | 1.40 | 12.84 | 13.55 | 6.26 | 6.41 | 13.26 | 3.50 | 5.64 | 3.49 |
| D046_pre | 0.03 | 0.02 | 0.06 | 0.04 | 0.03 | 0.08 | 0.05 | 1.23 | 0.09 | 0.25 | 0.57 | 0.54 |
| D046_post | 1.31 | 0.16 | 0.57 | 0.75 | 0.23 | 0.79 | 1.60 | >50 | 1.19 | 0.82 | 3.69 | 3.09 |
| D047_pre | 0.32 | 0.22 | 0.95 | 0.21 | 0.82 | 0.10 | 0.92 | 8.21 | 0.17 | 4.51 | 1.28 | 2.72 |
| D047_post | 17.05 | 3.64 | 4.36 | 4.71 | 24.24 | 3.05 | 13.78 | >50 | 16.13 | >50 | 9.53 | 8.73 |
| D048_pre | 0.04 | 0.03 | 0.07 | 0.08 | 0.50 | 0.84 | 0.03 | 16.75 | 0.36 | 0.49 | 1.40 | 0.07 |
| D048_post | 0.13 | 0.03 | 0.07 | 2.03 | 4.83 | 8.60 | 0.10 | >50 | 7.30 | 3.27 | 5.76 | 0.24 |
| D049_pre | 0.16 | 0.03 | 0.20 | 0.46 | 1.62 | 0.05 | 0.17 | 1.35 | 0.25 | 0.46 | 0.69 | 0.82 |
| D049_post | 8.01 | 0.11 | 1.04 | 1.32 | 12.02 | 0.28 | 1.32 | 9.32 | 1.32 | 0.78 | 4.72 | 1.33 |
| D050_pre | 0.03 | 0.04 | 0.06 | 0.39 | 0.04 | 0.96 | 0.05 | 1.00 | 0.05 | 0.45 | 0.31 | 0.13 |
| D050_post | 0.25 | 0.63 | 0.20 | 5.04 | 0.39 | 8.17 | 1.62 | 2.93 | 0.13 | 7.37 | 0.83 | 1.60 |
| D051_pre | 0.04 | 0.08 | 0.06 | 0.04 | 0.04 | 0.05 | 0.05 | 0.11 | 0.12 | 0.36 | 0.29 | 0.04 |
| D051_post | 0.44 | 0.13 | 0.51 | 0.11 | 0.28 | 0.09 | 0.24 | 3.75 | 0.31 | 0.48 | 0.73 | 0.12 |
| D052_pre | 0.09 | 0.17 | 0.38 | 0.21 | 0.42 | 0.12 | 0.24 | 0.86 | 0.32 | 1.90 | 1.37 | 11.84 |
| D052_post | 0.75 | 0.23 | 0.41 | 1.69 | 0.51 | 0.73 | 0.35 | >50 | 1.22 | 16.36 | 10.27 | >50 |
| D053_pre | 1.53 | 0.02 | 0.05 | 3.11 | 0.03 | 0.34 | 0.02 | 0.10 | 1.52 | 0.29 | 0.27 | 0.08 |
| D053_post | 19.33 | 0.55 | 0.24 | 15.59 | 0.21 | 6.73 | 0.43 | 0.13 | 21.37 | 9.01 | 4.64 | 0.35 |
| D055_pre | 1.01 | 0.60 | 0.99 | 0.23 | 0.12 | 1.29 | 0.65 | 2.05 | 0.15 | 1.16 | 0.77 | 1.34 |
| D055_post | 5.08 | 3.29 | 1.19 | 1.80 | 1.75 | 12.07 | 1.67 | >50 | 0.79 | 5.39 | 4.34 | 2.21 |
| D056_pre | 0.20 | 0.04 | 0.18 | 0.06 | 0.02 | 0.17 | 0.37 | 0.37 | 0.25 | 0.28 | 0.74 | 0.37 |
| D056_post | 22.82 | 0.06 | 0.40 | 1.94 | 0.04 | 2.64 | 0.66 | 4.77 | 6.28 | 0.39 | 7.82 | 16.39 |
| D057_pre | 0.13 | 0.98 | 0.81 | 0.13 | 0.18 | 1.24 | 0.58 | 2.25 | 0.57 | 12.25 | 1.10 | 1.21 |
| D057_post | 0.40 | 0.88 | 0.81 | 0.38 | 0.19 | 8.79 | 0.54 | 2.03 | 2.53 | 17.00 | 4.09 | 1.10 |
| D058_pre | 0.13 | 0.05 | 0.14 | 0.09 | 0.06 | 0.25 | 0.22 | 0.76 | 0.13 | 0.53 | 0.60 | 0.39 |
| D058_post | 1.14 | 0.09 | 0.17 | 0.24 | 0.63 | 1.23 | 0.30 | 1.20 | 0.12 | 0.96 | 0.74 | 0.26 |
| D059_pre | 0.02 | 0.51 | 0.10 | 0.02 | 0.02 | 0.26 | 0.03 | 2.87 | 0.26 | 0.27 | 0.44 | 0.08 |
| D059_post | 0.18 | 1.02 | 0.50 | 0.04 | 0.04 | 2.94 | 0.14 | 10.25 | 3.32 | 1.59 | 1.12 | 0.26 |
| D060_pre | 1.98 | 0.15 | 0.19 | 0.15 | 0.15 | 0.14 | 0.15 | 0.95 | 0.27 | 1.09 | 1.22 | 0.30 |
| D060_post | 24.99 | 0.23 | 0.66 | 0.89 | 1.04 | 2.88 | 3.09 | >50 | 2.76 | >50 | 6.22 | 0.91 |
| D061_pre | 1.19 | 0.69 | 0.25 | 0.16 | 0.09 | 0.99 | 0.14 | 0.52 | 1.92 | 0.78 | 1.41 | 0.42 |
| D061_post | 24.77 | 2.38 | 0.24 | 1.22 | 0.07 | 9.07 | 0.43 | 0.38 | 15.19 | 0.84 | 1.52 | 1.69 |
| D062_pre | 0.07 | 0.20 | 0.20 | 0.09 | 0.18 | 0.14 | 0.42 | 0.68 | 0.47 | 1.25 | 1.06 | 1.25 |
| D062_post | 5.09 | 1.15 | 7.94 | 0.98 | 16.13 | 3.28 | 7.21 | 2.86 | 1.86 | 13.05 | 19.59 | 29.33 |
| D063_pre | 0.06 | 0.17 | 0.18 | 0.05 | 0.12 | 0.35 | 0.63 | 0.49 | 0.53 | 10.38 | 0.52 | 0.37 |
| D063_post | 0.25 | 0.26 | 1.64 | 1.38 | 0.81 | 7.52 | 21.57 | 10.03 | 38.21 | >50 | 2.06 | 0.91 |
| D064_pre | 0.02 | 0.09 | 0.13 | 0.03 | 0.13 | 0.25 | 0.58 | 0.93 | 0.46 | 2.73 | 0.67 | 0.76 |
| D064_post | 0.37 | 0.93 | 1.16 | 0.49 | 4.45 | 4.89 | 6.93 | 22.04 | 3.85 | >50 | 6.06 | 2.05 |
| D066_pre | 0.78 | 0.19 | 0.97 | 4.89 | 0.45 | 0.62 | 0.75 | 3.79 | 1.68 | 2.89 | 4.75 | 2.31 |
| D066_post | 21.18 | 0.70 | 1.33 | 14.82 | 2.26 | 12.70 | 1.24 | 5.67 | 36.75 | 8.04 | 44.98 | 3.96 |
| D067_pre | 0.10 | 0.08 | 0.14 | 1.11 | 0.72 | 0.15 | 0.38 | 1.09 | 0.69 | 2.43 | 1.78 | 1.10 |
| D067_post | 0.78 | 0.23 | 0.93 | 15.73 | 2.07 | 3.42 | 1.04 | 12.31 | 5.72 | 8.22 | 8.06 | 1.58 |
| D068_pre | 0.16 | 0.14 | 0.26 | 0.13 | 0.09 | 0.05 | 0.15 | 0.49 | 0.18 | 1.03 | 0.84 | 1.69 |
| D068_post | 0.71 | 0.59 | 0.58 | 0.62 | 0.16 | 1.61 | 0.70 | 0.69 | 1.16 | 2.56 | 1.30 | 6.44 |
| D069_pre | 0.07 | 0.05 | 0.47 | 0.07 | 3.26 | 0.26 | 0.56 | 23.04 | 0.25 | 4.00 | 1.96 | 8.22 |
| D069_post | 0.94 | 0.18 | 2.14 | 0.37 | 41.38 | 10.13 | 8.64 | 34.43 | 3.18 | 35.03 | 3.13 | 40.21 |
| D070_pre | 0.08 | 0.07 | 0.05 | 0.05 | 0.50 | 1.45 | 0.05 | 0.09 | 0.24 | 1.55 | 0.55 | 0.49 |
| D070_post | 2.55 | 0.69 | 1.05 | 0.27 | 6.07 | 16.75 | 1.36 | 3.58 | 10.05 | 13.45 | >50 | 13.24 |
| D071_pre | 3.68 | 0.12 | 0.95 | 3.06 | 0.22 | 1.29 | 1.34 | 1.16 | 1.18 | 1.52 | 1.51 | 1.22 |
| D071_post | 23.38 | 0.35 | 2.85 | 14.20 | 5.59 | 7.58 | 24.03 | 31.06 | 48.98 | 2.88 | 13.83 | 25.79 |
| D072_pre | 0.07 | 2.19 | 0.10 | 1.53 | 0.05 | 0.44 | 0.14 | 0.31 | 0.09 | 0.70 | 0.51 | 4.97 |
| D072_post | 22.40 | 3.62 | 2.31 | 8.29 | 1.46 | 3.43 | 24.69 | 0.40 | 1.20 | 0.93 | 1.68 | 10.50 |
| D073_pre | 0.12 | 0.18 | 0.38 | 0.15 | 0.15 | 0.31 | 0.14 | 4.58 | 0.23 | 1.33 | 1.70 | 0.29 |
| D073_post | 5.91 | 0.28 | 0.72 | 0.30 | 0.20 | 1.74 | 0.50 | 13.68 | 1.71 | 3.92 | 4.23 | 0.76 |
| D074_pre | 0.19 | 0.14 | 1.17 | 0.46 | 0.16 | 0.10 | 0.89 | 12.03 | 0.65 | 2.15 | 3.40 | 1.94 |
| D074_post | 11.10 | 0.93 | 3.77 | 15.15 | 0.83 | 1.13 | 7.88 | >50 | 11.91 | 40.15 | >50 | 4.31 |
| D075_pre | 0.06 | 0.76 | 0.17 | 0.03 | 0.04 | 0.48 | 0.05 | 0.28 | 0.15 | 0.32 | 0.36 | 0.05 |
| D075_post | 2.73 | 15.22 | 8.53 | 1.10 | 10.71 | 13.19 | 1.76 | 0.85 | 12.60 | 6.38 | 5.21 | 2.12 |
| D076_pre | 0.03 | 0.38 | 0.25 | 0.09 | 1.82 | 0.15 | 0.21 | 5.07 | 0.86 | 0.38 | 1.16 | 0.42 |
| D076_post | 0.70 | 0.49 | 0.99 | 1.24 | >50 | 4.71 | 1.08 | >50 | 11.29 | 12.15 | 13.60 | 0.95 |
| D077_pre | 0.29 | 0.05 | 0.25 | 1.02 | 0.23 | 0.22 | 0.17 | 0.71 | 0.25 | 2.92 | 1.26 | 1.55 |
| D077_post | 17.67 | 0.96 | 0.61 | 18.20 | 10.91 | 3.80 | 2.66 | 3.45 | 5.01 | 22.02 | 26.84 | 9.08 |
| D078_pre | 0.14 | 0.01 | 0.06 | 0.10 | 0.32 | 0.01 | 0.05 | 0.16 | 0.94 | 8.83 | 2.72 | 0.72 |
| D078_post | 18.06 | 0.03 | 0.17 | 0.21 | 0.67 | 0.03 | 1.76 | 0.43 | 11.09 | 12.88 | 41.83 | 2.03 |
| D080_pre | 0.22 | 1.09 | 2.98 | 1.04 | 1.10 | 0.26 | 1.47 | 12.45 | 0.93 | 3.24 | 5.25 | 4.22 |
| D080_post | 0.91 | 6.79 | 5.70 | 6.45 | 20.30 | 1.16 | 3.33 | 17.33 | 5.53 | 9.55 | 15.64 | 6.70 |
| D081_pre | 1.61 | 0.30 | 0.59 | 2.05 | 0.40 | 0.68 | 0.50 | 2.73 | 1.07 | 12.83 | 4.31 | 0.77 |
| D081_post | 20.36 | 1.62 | 45.63 | 18.94 | 5.38 | 15.94 | 8.16 | 27.00 | 8.37 | 40.40 | 25.91 | 1.67 |
| D082_pre | 0.05 | 0.12 | 0.13 | 0.21 | 0.22 | 0.20 | 0.12 | 13.01 | 0.23 | 0.31 | 1.61 | 0.91 |
| D082_post | 1.64 | 0.13 | 0.86 | 1.43 | 22.85 | 0.46 | 0.67 | >50 | 2.77 | 0.78 | 5.95 | 9.66 |
| D083_pre | 0.05 | 0.07 | 0.13 | 0.06 | 0.05 | 0.05 | 0.13 | 2.13 | 0.16 | 4.90 | 0.64 | 0.74 |
| D083_post | 0.22 | 0.30 | 0.30 | 1.87 | 0.10 | 1.31 | 0.27 | 29.59 | 3.08 | 11.90 | 2.73 | 5.00 |
| D084_pre | 0.06 | 0.54 | 0.06 | 0.19 | 0.27 | 0.09 | 0.04 | 1.18 | 2.59 | 0.26 | 0.58 | 0.49 |
| D084_post | 1.82 | 2.13 | 0.10 | 0.45 | 43.54 | 1.66 | 0.22 | 6.28 | 13.70 | 1.17 | 3.48 | 7.64 |
| D085_pre | 0.03 | 0.05 | 0.09 | 0.11 | 0.02 | 0.03 | 0.04 | 0.42 | 0.16 | 1.31 | 0.81 | 0.16 |
| D085_post | 1.73 | 0.38 | 1.09 | 2.14 | 1.27 | 0.32 | 0.60 | 0.94 | 1.31 | 3.60 | 2.32 | 0.56 |
| D086_pre | 0.03 | 0.04 | 0.05 | 0.05 | 0.03 | 0.21 | 0.05 | 0.37 | 0.30 | 0.17 | 0.29 | 0.17 |
| D086_post | 2.31 | 0.17 | 0.91 | 1.64 | 1.70 | 9.09 | 3.21 | 2.15 | 11.78 | 1.84 | 1.35 | 1.08 |
| D087_pre | 0.14 | 3.59 | 1.05 | 0.15 | 0.45 | 0.65 | 2.15 | 10.61 | 2.70 | 7.92 | 1.70 | 1.65 |
| D087_post | 3.89 | 21.31 | 17.93 | 15.98 | 44.94 | 17.84 | >50 | >50 | 14.09 | 43.94 | 29.67 | 6.28 |
| D088_pre | 0.39 | 0.85 | 1.00 | 0.33 | 0.34 | 0.13 | 0.30 | >50 | 2.04 | 3.70 | 5.94 | 0.74 |
| D088_post | 8.73 | 0.64 | 1.08 | 1.11 | 2.85 | 0.45 | 1.13 | >50 | 3.25 | 28.32 | 14.47 | 1.17 |
| D089_pre | 1.51 | 0.11 | 0.11 | 0.07 | 0.06 | 0.39 | 0.19 | 0.55 | 0.10 | 0.42 | 1.11 | 0.20 |
| D089_post | 20.60 | 0.40 | 0.73 | 0.11 | 0.34 | 13.41 | 4.48 | 1.36 | 2.12 | 0.59 | 1.55 | 2.20 |
| D090_pre | 0.03 | 0.21 | 0.06 | 0.26 | 0.02 | 0.15 | 0.04 | 0.38 | 0.10 | 3.31 | 0.35 | 0.11 |
| D090_post | 0.53 | 3.30 | 0.83 | 3.18 | 0.47 | 4.26 | 1.89 | 12.78 | 2.11 | 14.11 | 2.22 | 2.34 |
| D091_pre | 0.36 | 1.31 | 0.34 | 0.16 | 1.87 | 0.16 | 0.22 | 2.16 | 0.13 | 1.38 | 1.73 | 0.78 |
| D091_post | 5.62 | 1.34 | 0.66 | 16.54 | 11.22 | 3.59 | 5.03 | >50 | 0.44 | 8.11 | 5.09 | 16.75 |
| D092_pre | 4.03 | 0.23 | 0.23 | 0.05 | 2.90 | 0.45 | 0.18 | 1.21 | 0.15 | 0.46 | 1.78 | 1.87 |
| D092_post | >50 | 10.07 | 5.05 | 0.70 | 49.46 | 27.94 | 2.51 | 39.01 | 8.24 | 1.09 | 7.96 | 8.10 |
| D093_pre | 2.78 | 0.54 | 0.19 | 0.53 | 0.20 | 0.69 | 0.24 | 8.44 | 0.21 | 2.19 | 2.43 | 0.42 |
| D093_post | 30.50 | 5.57 | 11.16 | 11.36 | 16.80 | 28.37 | 3.77 | >50 | 2.71 | 26.68 | 25.85 | 14.27 |
| D094_pre | 0.15 | 0.12 | 0.15 | 0.42 | 0.08 | 0.19 | 0.20 | 1.00 | 0.10 | 0.27 | 0.82 | 0.62 |
| D094_post | 17.16 | 1.53 | 2.09 | 30.80 | 1.65 | 3.41 | 3.29 | 0.94 | 2.62 | 2.42 | 6.46 | 14.20 |
| D095_pre | 0.08 | 0.07 | 0.07 | 0.03 | 0.10 | 0.04 | 0.09 | 0.29 | 0.05 | 3.59 | 0.73 | 0.26 |
| D095_post | 36.51 | 0.23 | 4.19 | 0.39 | 3.13 | 5.53 | 14.78 | 0.43 | 0.52 | >50 | 5.75 | 9.21 |
| D096_pre | 0.57 | 0.04 | 0.11 | 0.07 | 0.40 | 0.51 | 0.14 | 15.16 | 0.78 | 0.63 | 1.78 | 1.05 |
| D096_post | 14.98 | 0.16 | 0.25 | 0.65 | 6.62 | 6.12 | 0.30 | >50 | 10.44 | 0.73 | 4.84 | 6.49 |
| D097_pre | 0.18 | 0.04 | 0.63 | 0.22 | 0.23 | 1.09 | 0.22 | 4.08 | 0.23 | 2.43 | 5.03 | 0.25 |
| D097_post | 3.40 | 0.29 | 37.59 | 28.51 | 3.15 | 3.06 | 10.67 | 37.73 | 3.54 | 5.24 | 9.08 | 15.68 |
| D098_pre | 0.05 | 0.14 | 0.34 | 0.34 | 1.40 | 4.64 | 0.34 | 1.43 | 1.30 | 0.36 | 1.83 | 0.86 |
| D098_post | 0.68 | 0.22 | 4.16 | 7.90 | 7.69 | 20.93 | 2.76 | 15.47 | 11.15 | 0.82 | 17.05 | 5.99 |
| D099_pre | 0.42 | 0.32 | 0.63 | 0.14 | 0.16 | 0.06 | 0.22 | 1.33 | 0.13 | 1.00 | 2.09 | 1.18 |
| D099_post | 10.87 | 0.63 | 1.42 | 0.58 | 0.59 | 0.59 | 0.70 | 1.42 | 0.72 | 2.12 | 2.63 | 6.60 |
| D100_pre | 0.06 | 0.04 | 0.19 | 0.11 | 0.20 | 1.05 | 0.17 | 0.34 | 0.16 | 2.27 | 2.66 | 0.13 |
| D100_post | 0.46 | 0.29 | 3.67 | 2.40 | 9.12 | 2.75 | >50 | 0.90 | 0.99 | >50 | >50 | 4.12 |
| D101_pre | 0.04 | 0.94 | 0.19 | 0.30 | 0.05 | 0.17 | 0.10 | 0.88 | 0.50 | 22.81 | 0.54 | 0.23 |
| D101_post | 0.11 | 1.17 | 14.48 | 0.77 | 0.11 | 0.81 | 3.65 | 2.20 | 4.41 | >50 | 0.71 | 0.53 |
| D102_pre | 0.28 | 0.11 | 1.19 | 0.46 | 0.60 | 4.00 | 0.51 | 3.10 | 0.34 | 2.03 | 8.85 | 7.55 |
| D102_post | 1.06 | 0.24 | 1.78 | 20.29 | 27.93 | 19.64 | 1.60 | 12.44 | 8.16 | 2.72 | >50 | 19.41 |
| D103_pre | 0.03 | 0.10 | 0.17 | 0.04 | 2.80 | 0.09 | 0.17 | 3.27 | 0.18 | 10.64 | 1.44 | 0.56 |
| D103_post | 0.13 | 0.40 | 0.35 | 0.36 | 34.20 | 3.40 | 1.73 | 30.62 | 4.31 | >50 | 27.47 | 3.95 |
| D104_pre | 0.07 | 0.03 | 0.04 | 0.02 | 0.13 | 0.46 | 0.05 | 0.25 | 0.49 | 2.37 | 0.51 | 0.17 |
| D104_post | 0.48 | 0.26 | 0.23 | 0.23 | 0.56 | 27.75 | 0.77 | 8.26 | 5.25 | 24.00 | 3.48 | 0.67 |
| D105_pre | 0.23 | 0.03 | 0.14 | 0.02 | 0.04 | 0.46 | 0.06 | 0.21 | 1.10 | 0.27 | 0.69 | 0.05 |
| D105_post | 0.39 | 0.12 | 0.70 | 0.09 | 0.12 | 4.20 | 0.38 | 8.35 | 1.51 | 0.61 | 2.50 | 2.10 |
| D106_pre | 0.27 | 0.63 | 0.83 | 1.79 | 7.20 | 7.03 | 0.61 | 4.84 | 0.84 | 3.97 | 6.45 | 1.90 |
| D106_post | 5.00 | 3.27 | 2.42 | 29.64 | >50 | 34.93 | 13.98 | 6.15 | 16.95 | >50 | >50 | 9.44 |
| D107_pre | 0.04 | 0.13 | 0.40 | 0.76 | 0.33 | 0.31 | 0.71 | 3.02 | 0.81 | 2.02 | 1.66 | 0.71 |
| D107_post | 0.21 | 1.53 | 0.50 | 22.96 | 0.43 | 1.46 | 9.49 | 3.10 | 17.16 | 10.45 | 10.31 | 0.58 |
| D108_pre | 0.10 | 0.12 | 0.11 | 0.07 | 0.04 | 0.43 | 0.09 | 1.68 | 0.18 | 0.95 | 0.69 | 0.14 |
| D108_post | 0.43 | 0.13 | 0.30 | 3.77 | 0.17 | 1.91 | 0.43 | 4.34 | 0.84 | 1.05 | 3.69 | 0.61 |
| D109_pre | 1.10 | 0.15 | 0.38 | 0.47 | 0.25 | 0.13 | 0.20 | 1.08 | 0.15 | 0.60 | 1.61 | 0.59 |
| D109_post | 20.70 | 0.42 | 2.90 | 4.15 | 4.67 | 0.47 | 2.14 | 1.48 | 4.47 | 0.56 | 2.99 | 21.99 |
| D110_pre | 0.04 | 0.09 | 0.11 | 0.12 | 0.10 | 0.04 | 0.21 | 28.84 | 0.30 | 4.45 | 2.61 | 0.11 |
| D110_post | 0.55 | 0.24 | 0.21 | 0.29 | 0.15 | 0.09 | 1.21 | 40.24 | 1.97 | 24.42 | 9.72 | 0.16 |
| D111_pre | 0.19 | 0.21 | 0.38 | 0.50 | 1.75 | 0.23 | 0.21 | 4.33 | 1.73 | 2.61 | 2.37 | 0.34 |
| D111_post | 8.80 | 0.37 | 0.99 | 2.29 | 43.09 | 1.60 | 1.05 | 5.83 | 8.11 | 27.20 | 16.79 | 0.38 |
| D113_pre | 0.18 | 0.11 | 0.29 | 0.24 | 0.42 | 0.08 | 0.50 | 5.78 | 0.12 | 0.78 | 1.73 | 0.19 |
| D113_post | 0.88 | 0.40 | 0.62 | 2.72 | 1.84 | 1.67 | 9.64 | 5.12 | 0.56 | 4.00 | 1.62 | 0.15 |
| D114_pre | 0.10 | 0.02 | 0.32 | 0.03 | 0.03 | 0.07 | 0.13 | 0.25 | 0.20 | 3.04 | 0.33 | 0.07 |
| D114_post | 2.49 | 0.03 | 1.14 | 0.54 | 0.35 | 0.21 | 1.18 | 3.72 | 11.15 | >50 | 2.10 | 0.10 |
| D115_pre | 0.05 | 0.33 | 0.66 | 0.29 | 0.11 | 0.40 | 0.21 | 0.61 | 1.76 | 1.41 | 1.38 | 0.13 |
| D115_post | 8.23 | 0.73 | 6.70 | 6.97 | 0.99 | 9.26 | 1.65 | 11.30 | 11.80 | 33.75 | 20.50 | 3.87 |
| D116_pre | 0.24 | 0.50 | 0.20 | 0.33 | 1.56 | 1.27 | 0.23 | 0.64 | 7.01 | 3.15 | 2.58 | 0.33 |
| D116_post | 0.27 | 6.58 | 0.36 | 0.43 | 8.51 | 15.36 | 4.70 | 2.40 | 12.03 | 37.45 | 24.17 | 0.76 |
| D117_pre | 0.07 | 0.03 | 0.47 | 0.18 | 0.37 | 0.05 | 0.16 | 2.31 | 0.29 | 0.75 | 0.72 | 0.47 |
| D117_post | 0.99 | 0.03 | 2.17 | 0.32 | 2.51 | 0.66 | 0.80 | 15.07 | 0.53 | 1.36 | 1.55 | 1.04 |
| D118_pre | 0.06 | 0.05 | 0.11 | 3.13 | 0.24 | 0.16 | 0.77 | 2.78 | 0.57 | 0.96 | 0.74 | 0.99 |
| D118_post | 0.47 | 0.48 | 0.35 | 18.76 | 1.56 | 0.70 | 2.75 | 4.72 | 5.91 | 11.49 | 1.62 | 6.71 |
| D119_pre | 0.02 | 0.06 | 0.06 | 0.02 | 0.02 | 0.25 | 0.06 | 0.42 | 0.07 | 0.10 | 0.23 | 0.04 |
| D119_post | 0.09 | 0.14 | 1.01 | 0.55 | 0.11 | 3.23 | 0.83 | 6.34 | 0.94 | 0.18 | 1.20 | 0.04 |
| D120_pre | 1.82 | 0.19 | 0.16 | 0.32 | 0.98 | 0.53 | 0.38 | 4.16 | 0.21 | 1.98 | 2.12 | 0.56 |
| D120_post | 2.07 | 1.47 | 1.07 | 9.36 | 15.56 | 10.57 | 16.47 | 10.08 | 6.87 | 12.55 | >50 | 7.61 |
| D121_pre | 0.05 | 0.03 | 0.10 | 0.08 | 0.03 | 0.03 | 0.04 | 0.30 | 0.10 | 0.30 | 0.55 | 0.13 |
| D121_post | 1.34 | 0.46 | 0.49 | 0.29 | 0.07 | 1.50 | 0.28 | 3.84 | 0.56 | 0.30 | 0.91 | 0.44 |
| D122_pre | 0.12 | 0.08 | 0.12 | 0.07 | 0.08 | 3.45 | 0.05 | 1.35 | 0.24 | 4.57 | 0.62 | 0.08 |
| D122_post | 0.28 | 0.09 | 0.71 | 0.91 | 0.18 | 3.68 | 0.71 | 14.61 | 0.61 | 12.39 | 8.33 | 0.15 |
| D123_pre | 0.03 | 0.51 | 0.19 | 0.05 | 0.04 | 0.03 | 0.12 | 1.02 | 0.09 | 0.60 | 0.98 | 0.45 |
| D123_post | 0.45 | 0.64 | 1.32 | 0.17 | 0.07 | 0.56 | 1.21 | 4.86 | 0.37 | 46.33 | 4.75 | 0.91 |
| D124_pre | 0.03 | 0.02 | 0.05 | 0.17 | 0.03 | 0.03 | 0.02 | 1.20 | 0.12 | 0.14 | 0.41 | 0.05 |
| D124_post | 0.40 | 0.04 | 0.14 | 9.83 | 0.20 | 4.00 | 0.59 | 2.33 | 0.61 | 0.45 | 2.40 | 0.72 |

### Supplementary Figure 1. **Scatter Plot of Antibody Concentrations Pre- and Post-Vaccination.**


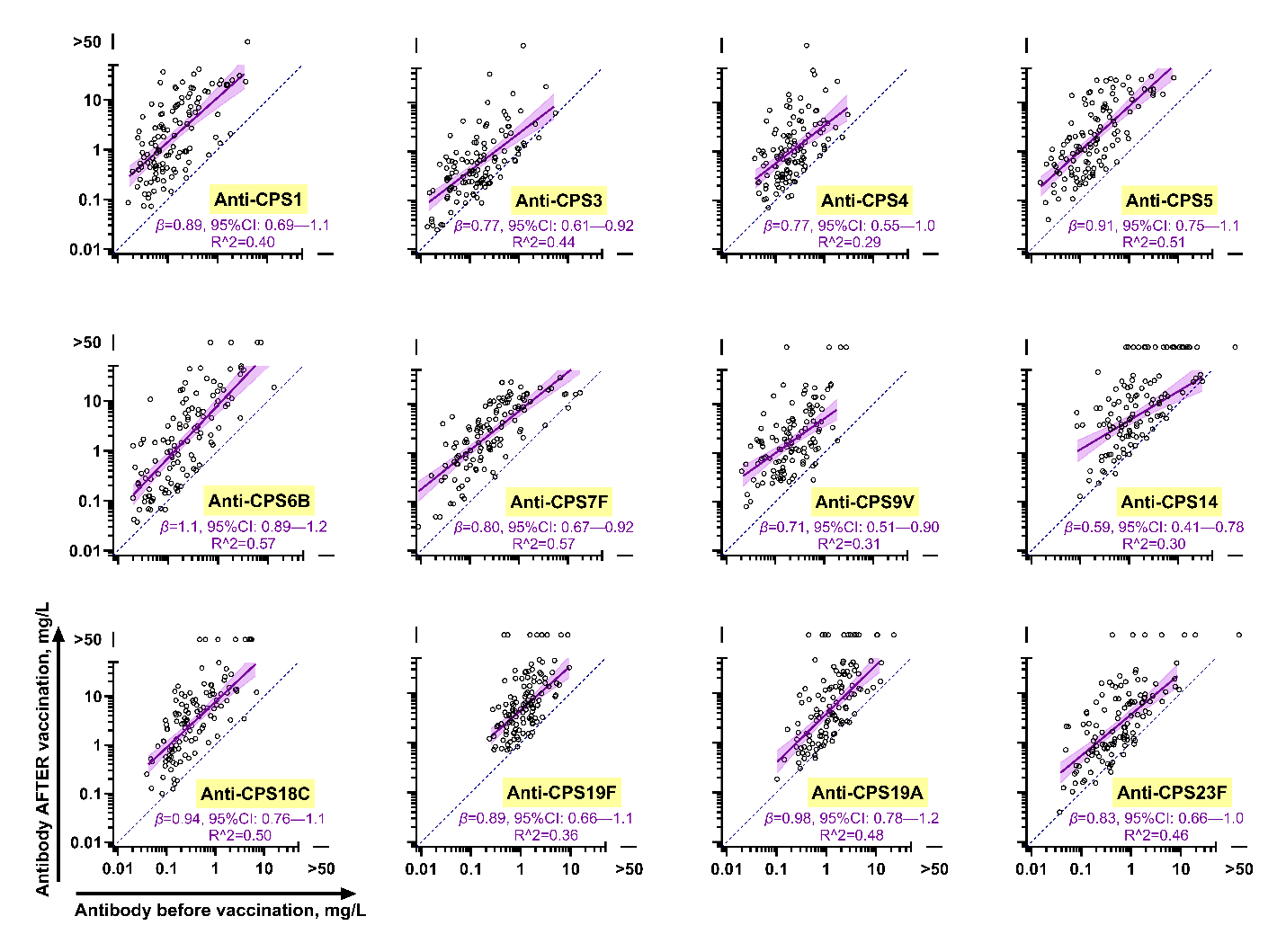


Scatter plot representation of concentrations for each of the 12 antibodies measured in study participants. Concentrations measured in samples collected before vaccination are depicted on the X-axis, while concentrations measured in samples collected after vaccination are shown on the Y-axis. Log-log transformed data is presented with antilog numbers on the axes.

Results of linear regression analyses are presented in purple. Outliers (measurements above 50 mg/L) were omitted from these analyses. Solid lines represent the regression curves, while the lighter area indicates the 95% confidence intervals. Slope estimates are provided with 95% confidence limits, as well as estimates of R².

### Supplementary Figure 2. Selection Process for Generating Antibody Sets.

###
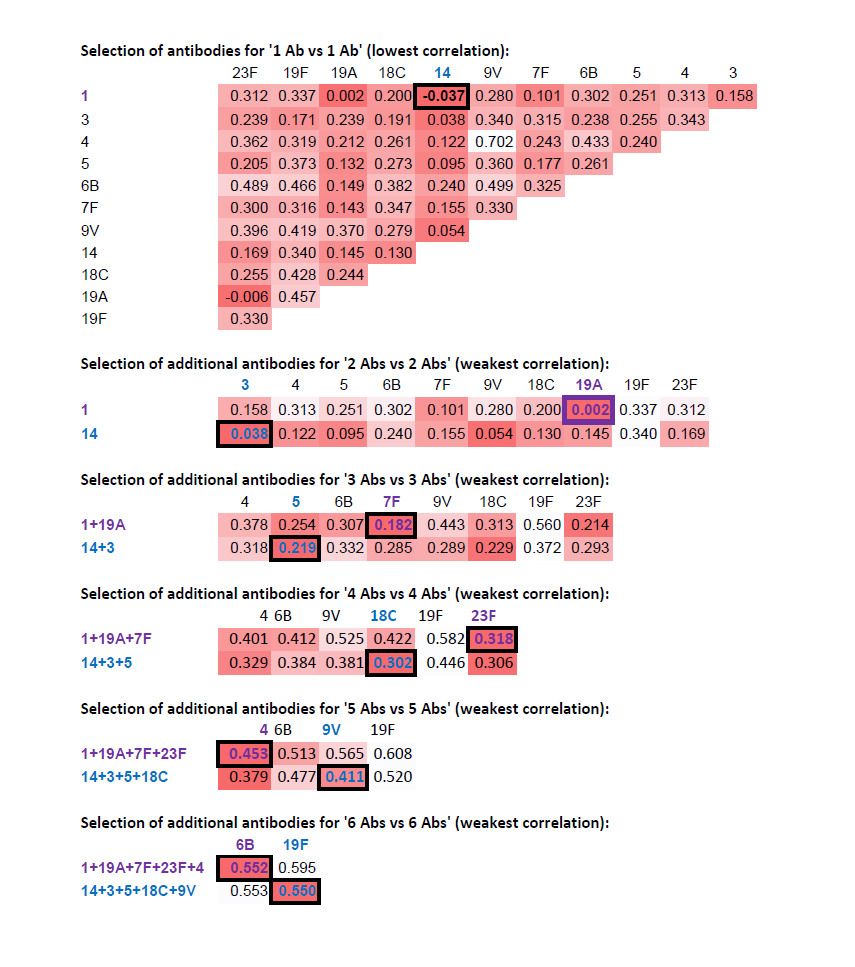


To examine antibody concentrations, expressed as percentiles, and their correlations, including the effect of combining measurements of different antibodies, we applied a conservative approach.

Scatter Plot of Two Individual Antibodies: We selected the two antibodies out of the twelve available that displayed the lowest Pearson correlation coefficient. The percentiles for these two antibodies, anti-CPS1 and anti-CPS14, are presented in **Figure 4**, labeled '1 Ab vs 1 Ab'.

Scatter plot of combined percentiles of two pairs of antibodies: Among the remaining ten antibodies, we identified those that showed the weakest correlation with anti-CPS1 and anti-CPS14. Anti-CPS1 was paired with anti-CPS19A, and anti-CPS14 was paired with anti-CPS3. For each pair, the percentiles were combined by summation and then expressed as a percentile relative to similar pairs for all participants. These combined percentiles are presented in **Figure 5**, labeled '2 Abs vs 2 Abs'.

Scatter plot of combined percentiles of two sets of three antibodies: From the remaining eight antibodies, we identified those with the weakest correlation to each of the pairs from the previous step. The percentiles of anti-CPS1 and anti-CPS19A were combined with those of anti-CPS7F. The percentiles of anti-CPS14 and anti-CPS3 were combined with those of anti-CPS5. These combined percentiles are presented in **Figure 5**, labeled '3 Abs vs 3 Abs'.

Similar procedures were followed to generate combined percentiles for two sets of four antibodies, two sets of five antibodies, and two sets of six antibodies. These combinations are presented in **Figure 5**, labeled '4 Abs vs 4 Abs', '5 Abs vs 5 Abs', and '6 Abs vs 6 Abs', respectively.
